# miR-512-3p/RPS6KA2 Axis Regulates Cisplatin Resistance in Ovarian Cancer via Autophagy and Ferroptosis

**DOI:** 10.32604/or.2025.070542

**Published:** 2025-12-30

**Authors:** Jianfa Wu, Huang Chen, Sihong Wang, Lei Peng, Xiaoying Hu, Zhou Liu

**Affiliations:** 1 Department of Gynecology, Shanghai University of Medicine & Health Sciences Affiliated Zhoupu Hospital, Shanghai, 201318, China; 2 Department of Gynecology, Shanghai University of Medicine & Health Sciences, Shanghai, 201318, China; 3 Department of Integrated Traditional Chinese and Western Medicine Clinical Practice, Shanghai University of Traditional Chinese Medicine, Shanghai, 200032, China

**Keywords:** RPS6KA2, autophagy, ovarian cancer, ferroptosis, chemoresistance

## Abstract

**Objectives:**

Ribosomal protein S6 kinase A2 (RPS6KA2) has been identified as a potential prognostic biomarker in several cancers, including breast cancer, glioblastoma, and prostate cancer. However, its functional significance in ovarian cancer is not well characterized. This study was designed to explore the therapeutic relevance of modulating RPS6KA2 in the context of ovarian cancer, particularly in relation to cisplatin resistance.

**Methods:**

The expression levels of RPS6KA2 and key regulators involved in autophagy and ferroptosis were assessed using quantitative reverse transcription-PCR, immunofluorescence staining, immunohistochemistry, and western blotting. Prognostic associations were conducted using the Kaplan-Meier Plotter database. Autophagy flux assays and visualization of autophagosomes were performed to assess autophagy activity. Ferroptosis-related parameters, including intracellular iron content, glutathione (GSH) levels, reactive oxygen species (ROS) generation, and mitochondrial membrane potential, were measured to determine ferroptotic changes. *In vivo* experiments were carried out to determine the antitumor efficacy of RPS6KA2 modulation in combination with pathway-specific agents.

**Results:**

Using ovarian cancer cell lines and clinical tissue samples, we demonstrated that RPS6KA2 expression was significantly downregulated in cisplatin-resistant cells and tissues compared to their sensitive counterparts. Low RPS6KA2 expression correlated with unfavorable patient outcomes and enhanced chemoresistance. Mechanistically, RPS6KA2 inhibited autophagy by modulating the phosphatidylinositol 3-kinase-protein kinase B-mammalian target of rapamycin (PI3K-AKT-mTOR) signaling pathway, which in turn increased sensitivity to cisplatin. Additionally, RPS6KA2 facilitated ferroptosis, contributing to its tumor-suppressive function. miR-512-3p was identified as a negative regulator of RPS6KA2, driving cisplatin resistance through suppression of RPS6KA2 expression. *In vivo* validation confirmed that combining RPS6KA2 targeting with autophagy inhibitors or ferroptosis inducers significantly enhanced cisplatin sensitivity in ovarian cancer models.

**Conclusion:**

These results collectively indicate that targeting the miR-512-3p/RPS6KA2 regulatory axis may offer a novel and effective strategy for overcoming cisplatin resistance in ovarian cancer.

## 1 Introduction

Ovarian cancer ranks among the most aggressive malignancies affecting the female reproductive system, primarily due to difficulties in early detection and the frequent development of treatment resistance in advanced stages [1,2]. Despite a favorable initial response rate to standard therapy involving cytoreductive surgery and platinum-based chemotherapy, more than 70% of patients relapse within two years, and the overall five-year survival rate for advanced cases remains below 50% [3]. Following relapse, the onset of platinum resistance significantly limits therapeutic options [4,5]. Second-line treatments, including chemotherapeutic agents such as liposomal doxorubicin and gemcitabine, as well as targeted therapies like PARP inhibitors (e.g., olaparib and niraparib), often lose effectiveness as resistance mechanisms emerge [6,7]. Although poly(ADP-ribose) polymerase (PARP) inhibitors demonstrate strong clinical benefit in patients with BRCA1 DNA repair-associated (BRCA) mutations, their limited efficacy in patients without these genetic alterations underscores a persistent and significant challenge in clinical oncology.

Resistance to cisplatin in ovarian cancer arises through a highly intricate and multifaceted network of biological alterations. Key mechanisms involve the abnormal expression of drug efflux proteins such as P-glycoprotein, dysregulation of copper transporters [8], alterations in DNA repair pathways [9], and disruptions in critical signaling pathways including autophagy and Nrf2 signaling pathways [10–12]. Additionally, changes in the tumor microenvironment have been shown to play a significant role in promoting chemoresistance [13]. Recent studies have highlighted the growing importance of mitochondrial metabolic dysregulation, imbalances in iron metabolism, and epigenetic regulation in driving resistance to cisplatin in ovarian cancer [14–16]. However, because these resistance mechanisms vary substantially among individuals, identifying a broadly applicable therapeutic target to counteract cisplatin resistance remains a major challenge in clinical practice.

The ribosomal protein S6 kinase A2 (RPS6KA2) functions as a tumor suppressor gene and is commonly subject to deletion or downregulation, with studies identifying its abnormal expression as a potential marker of poor prognosis in breast cancer, glioblastoma, and prostate cancer [17–19]. Reduced expression of RPS6KA2 has been linked to increased apoptosis in pancreatic cancer cells when exposed to erlotinib [20]. Accumulating data further suggest that RPS6KA2 plays a role in the emergence of resistance to tamoxifen in breast cancer, as well as to various other targeted treatments [21,22]. Additional research indicates its involvement in the integration of HPV16 viral sequence into the host genome, a process that may contribute to the development of anal cancer [23]. In ovarian cancer, recent findings show that RPS6KA2 expression is regulated by the non-coding RNA networks involving circFAM169A/miR-106a-5p and miR-519d-3p, which in turn influence the proliferative capacity of ovarian cancer cells [24].

This study seeks to explore the function of RPS6KA2 in reversing cisplatin resistance in ovarian cancer through comprehensive *in vivo* and *in vitro* approaches. Furthermore, the potential of RPS6KA2 as a therapeutic target for mitigating cisplatin resistance in ovarian cancer is rigorously assessed.

## 2 Material and Methods

### 2.1 Reagents and Cells

The human ovarian cancer cell lines A2780 and its cisplatin-resistant variant A2780CP were obtained from the American Type Culture Collection (ATCC, Manassas, VA, USA) and authenticated by short tandem repeat (STR) analysis to ensure adherence to human cell line DNA fingerprinting standards, with confirmation of mycoplasma-free status. The COC1 cell line and its cisplatin-resistant derivative COC1/DDP were acquired from Peking Union Medical College (Beijing, China) and similarly authenticated by STR analysis, fulfilling genetic identity criteria and testing negative for mycoplasma contamination. Syngeneic murine ovarian surface epithelial (MOSE) cells, originally isolated from C57BL/6 mice, underwent spontaneous malignant transformation during extended *in vitro* propagation. These phenotypically heterogeneous MOSE populations displayed progressive phenotypic heterogeneity, with morphological and functional shifts occurring across passages. Low-passage cells maintained a non-tumorigenic, premalignant phenotype, while mid-passage cultures showed intermediate characteristics. In contrast, late-passage variants demonstrated aggressive tumorigenicity and metastatic potential when transplanted into immunocompetent mice, indicative of complete neoplastic conversion. MOSE cell lines were authenticated by STR analysis and confirmed to be free of mycoplasma contamination. Cisplatin was procured from Aladdin (Shanghai, China). Honokiol (RPS6KA2 activator) and Erastin (ferroptosis inducer) were obtained from MedChemExpress (Monmouth Junction, NJ, USA). 3-Methyladenine (3-MA), employed as an autophagy inhibitor, was sourced from Sigma-Aldrich (Darmstadt, Germany).

### 2.2 Quantitative Reverse Transcription-PCR Analysis

The RNA concentration and purity were determined using a UV spectrophotometer (UV-3600, Shimadzu, Kyoto, Japan) after cell lysis (A2780, A2780CP, COC1, and COC1/DDP) with Trizol reagent (Invitrogen, Carlsbad, CA, USA). Total mRNA was then reverse-transcribed into complementary DNA (cDNA) using reverse transcriptase. Subsequently, qRT-PCR was performed in a reaction mixture containing cDNA template, gene-specific primers (Table 1), dNTPs, DNA polymerase, and SYBR Green fluorescent dye. The thermal cycling conditions consisted of an initial denaturation at 95°C for 30 s, followed by 39–40 cycles of denaturation at 95°C for 5 s and annealing/extension at 60°C for 30 s. A final melting curve analysis was carried out to verify the specificity of the amplified products. Relative gene expression levels were calculated using the 2^−ΔΔCt^ method based on the obtained data. All experiments were independently repeated in triplicate to ensure the reliability and reproducibility.

**Table 1:** Primers for genes

**Gene**	**Primers (F, 5^′^-3^′^)**	**Primers (R, 5^′^-3^′^)**
ATG5	TTGAATATGAAGGCACACCACTGA TCG	GTACTGTGATGTTCCAAGGAAGAGC
ATG7	GATGAATGAGCCTCCAACCT	GCAGCAATGTAAGACCAGTCAAGTC
BECN1	CACATCTGGCACAGTGGACAGT	GCATGGAGCAGCAACACAGTCT
BNIP3	GCATGAGTCTGGACGGAGTAGC	GCTCTGTTGGTATCTTGTGGTGTCT
GAPDH	AGATCATCAGCAATGCCTCCT	TGAGTCCTTCCACGATACCAA
LC3	ATGCCGTCGGAGAAGACCTTCA	TGGTTGGATGCTGCTCTCGAATAAG
MTOR	CGTCAGCACCATCAACCTCCAA	TCAGCCGTCTCAGCCATTCCA
SQSTM1	TGGCGGAGCAGATGAGGAAGA GGACTCCTAC	GGACTGGAGTTCACCTGTAGACG
RHEB	GATCCAACCATAGAA	CGGCTGTGTCTACAAGTTGAAGATG
RPS6KA2	ACGGAGGAGGATGTCAAGTTCTACC	CCAGGAGGATGTTCTCAGGCTTCA
TSC1	CCATCGACACGGCTGATAACTGAAC	CAGGAGAAGTTGGAGGAGTGGTCAT
TSC2	CTCTCAGGAACTCGCCGACATCT	TGGACAGGACGATCTCATAGGACAC
ULK1	TCTGCCTGTCGTCCACTGTGAA	GTGATGCTGTGAATGCGGTCCA

### 2.3 Cell Immunofluorescence Analysis

Cell immunofluorescence analysis was performed following a previously described protocol [13]. Ovarian cancer cells (A2780 and A2780CP) were seeded into 24-well plates at a density of 1 × 10^4^ cells per well. A2780 cells were divided into five experimental groups: negative control (NC), Cisplatin treatment (Cis), sh-RPS6KA2 + Cis, miR-512-3p mimics + Cis, and miR-512-3p mimics + Cis + RPS6KA2. A2780CP cells were also assigned to five groups: NC, cisplatin (Cis), RPS6KA2 + Cis, miR-512-3p inhibitor + Cis, and miR-512-3p inhibitor + Cis + sh-RPS6KA2. When cells reached 30%–50% confluence, they were fixed with 4% paraformaldehyde for 15 min, permeabilized with Triton X-100, and incubated at room temperature for 20 min. Subsequently, cells were blocked with bovine serum albumin (BSA) for 60 min, followed by incubation with primary antibodies against MTOR (66888-1-Ig, Proteintech, Chicago, IL, USA, diluted 1:500) or RHEB (15924-1-AP, Proteintech, USA, 1:100) for overnight incubation at 4°C. The next day, an Alexa Fluor 488-conjugated secondary antibody (A11008, Invitrogen, Carlsbad, CA, USA, diluted 1:1000) was applied and incubated at room temperature for 60 min in the dark. Finally, nuclei were stained with 4^′^, 6-diamidino-2-phenylindole (DAPI) for 15 min, and the samples were mounted with 10 μL of an anti-fade mounting medium to complete the procedure. All experiments were independently repeated in triplicate to ensure the reliability and reproducibility.

### 2.4 Survival Analysis

Survival analysis was evaluated through the Kaplan Meier plotter database (https://kmplot.com/analysis/ (accessed on 01 November 2025)), a publicly available tool that combines gene expression data with clinical survival information from three independent repositories: the Gene Expression Omnibus (GEO) dataset (https://www.ncbi.nlm.nih.gov/geo/ (accessed on 01 November 2025)), the European Genome-phenome Archive (EGA) dataset (https://ega-archive.org/ (accessedon 01 November 2025)), and the Cancer Genome Atlas (TCGA) dataset (https://portal.gdc.cancer.gov/ (accessedon 01 November 2025)). These integrated datasets provide expression data for around 54,000 genes across 21 different types of cancer. To determine the prognostic value of gene expression levels in cancer patients, data processing and analysis were carried out using a PostgreSQL server (Version 13). The log-rank test was applied to compute hazard ratios, while *p*-values and 95% confidence intervals were used to assess statistical significance [25].

### 2.5 High-Throughput Sequencing Analysis and Kyoto Encyclopedia of Genes and Genomes (KEGG) Enrichment Analysis

Ovarian tumor STAR-counts data along with matched clinical information from the TCGA database. The raw counts were transformed into transcripts per million (TPM) format and and further normalized using a log2(TPM + 1) transformation to reduce variability. Only those samples with both RNA-seq data and comprehensive clinical records were included in the analysis, yielding a final dataset of 188 samples for subsequent processing. Differential expression analysis of mRNA was performed using the ‘Limma’ package (version 3.40.2) in R software (version 4.5.0), and the results were visualized as a heatmap. To control for false discovery due to multiple comparisons, *p*-values were corrected using an adjustment method, and differentially expressed mRNAs were defined by the criteria: adjusted *p*-value < 0.05 with |log2(fold change)| > 1. Additionally, KEGG pathway enrichment analysis was conducted on the significantly expressed mRNAs using the ‘ClusterProfiler’ (Version 4.12.1) package in R [26].

### 2.6 Cell Proliferation Analysis

For the experimental setup, 5 × 10^3^ ovarian cancer cells (A2780 and A2780CP) were seeded into individual wells of 96-well plates. To investigate the role of RPS6KA2/TSC1/TSC2 in modulating cisplatin sensitivity in ovarian cancer, A2780 cells were assigned to five groups: negative control (NC), Cisplatin (5 μg/mL), sh-RPS6KA2 + Cisplatin, sh-RPS6KA2 + TSC1 + Cisplatin, and sh-RPS6KA2 + TSC2 + Cisplatin. Meanwhile, A2780CP cells were divided into five corresponding groups: NC, Cisplatin (20 μg/mL), RPS6KA2 + Cisplatin, RPS6KA2 + sh-TSC1 + Cisplatin, and RPS6KA2 + sh-TSC2 + Cisplatin. To explore the involvement of autophagy in cisplatin resistance, A2780 cells were further separated into four groups: negative control (NC), cisplatin alone, rapamycin combined with cisplatin treatment, and 3-MA combined with cisplatin. After a 24-h incubation, 20 μL of Cell Counting Kit-8 (CCK-8) reagent (CK04, Dojindo, Tokyo, Japan) was added to each well. The plate was then incubated for 2 h at 37°C in a humidified atmosphere containing 5% CO_2_. Thereafter, absorbance at 450 nm (ΔOD_450_) was recorded using a microplate reader (Model 3550, Hercules, CA, USA). All assays were independently repeated three times to ensure consistency and reproducibility of the findings.

### 2.7 Transmission Electron Microscopy (TEM)

Ovarian cancer cells (A2780 and A2780CP) were transfected with either plasmid or siRNA for 6 h according to the protocol outlined in a prior study [12]. After incubation for 48 h, a standardized cell count of 200,000 (2 × 10^5^) was collected and processed for transmission electron microscopy (TEM) according to established methods referenced in previous research [13]. Ultrathin sections of the samples were subsequently analyzed using a JEOL JEM-1400 transmission electron microscope (JEOL Ltd., Tokyo, Japan). All procedures were independently repeated three times to guarantee consistency, reliability and reproducibility of the experimental outcomes.

### 2.8 Subcutaneous Tumor Model in Nude Mice

A subcutaneous xenograft tumor model was established using female nude mice aged 6 to 8 weeks with a body weight of 18 to 20 g, which were acquired from the Shanghai Cancer Institute (Shanghai, China). The study utilized several ovarian cancer cell lines: A2780, A2780CP, A2780-sh-RPS6KA2, and A2780CP-RPS6KA2. To explore the role of the miR-512-3p/RPS6KA2 signaling axis in mediating cisplatin resistance, animals were randomly allocated into two sets of five groups (n = 6 per group) using computer-generated randomization sequences. The first set included: normal control (NC), cisplatin treatment (Cis), sh-RPS6KA2 + Cis, miR-512-3p mimics + Cis, and miR-512-3p mimics + Cis + sh-RPS6KA2; the second set consisted of NC, Cis, RPS6KA2 + Cis, miR-512-3p inhibitor + Cis, and miR-512-3p inhibitor + Cis + RPS6KA2. To evaluate the therapeutic targeting potential of RPS6KA2, an additional *in vivo* experiment was performed where mice were randomly assigned to 11 groups (n = 6 per group) via computer-based randomization: NC, Honokiol, Erastin, 3-MA, Honokiol + Cis, Erastin + Cis, 3-MA + Cis, Honokiol + Erastin + Cis, Honokiol + 3-MA + Cis, Erastin + 3-MA + Cis, and Honokiol + Erastin + 3-MA + Cis. Each mouse was injected subcutaneously with 1 × 10^8^ cells. Only those that developed measurable subcutaneous tumors were retained for further analysis, while non-tumor-forming individuals were excluded. Once the tumor volume reached approximately 300 mm^3^, mice received intraperitoneal injections of cisplatin (3.5 mg/kg/day), Honokiol (30 μg/kg), 3-MA (15 mg/kg), or Erastin (50 mg/kg) on days 0, 7, and 14 of the experiment. Concurrently, synthetic miR-512-3p mimic or inhibitor sequences (80 mg/kg), along with their respective negative controls (supplied by Shuoyan Biotechnology Co., Shanghai, China), were administered daily via tail vein injection over a 10-day period. Tumor sizes were measured weekly, and final comparisons were conducted after three weeks of treatment. To minimize bias, a blinded experimental design was implemented: one researcher administered treatments based on randomized codes and remained uninvolved in subsequent assessments; a second investigator performed surgical procedures and tumor excision; a third independently measured tumor dimensions using the formula V = (length × width^2^)/2 without knowledge of group assignments. Statistical differences between two groups were analyzed using an independent two-sample Student’s *t*-test with SPSS 22.0 software. All animal experiments were carried out in compliance with the National Research Council’s Guide for the Care and Use of Laboratory Animals and adhered to the ARRIVE guidelines. The study protocol was approved by Zhoupu Hospital Ethics Committee (Approval Number: 2025-C-210-E01).

### 2.9 Luciferase Reporter Assay

Luciferase reporter vectors harboring either the wild-type (WT) or mutant 3^′^ untranslated region (UTR) of RPS6KA2 were constructed by cloning PCR-amplified DNA fragments into the pmiR-RB-REPOR™ vector (RiboBio Co., Ltd., Guangzhou, China). Subsequently, cells (approximately 1 × 10^5^ per well in 24-well plates) were co-transfected with miR-512-3p mimics and either the WT (5^′^ UGCCUCGCUCCUGACUCAGCACUU 3^′^) or mutant RPS6KA2 3^′^ UTR (5^′^ UGCCUCGCUCCUGACCAGCACUU 3^′^) using Lipofectamine 2000 (1.5 mL, Invitrogen, Carlsbad, CA, USA) as the transfection reagent. Forty-eight hours after transfection, luciferase activity was assessed using the Dual-Luciferase® Reporter Assay System (E1910, Promega, Madison, WI, USA) according to the manufacturer’s protocol [27]. All assays were independently repeated three times to ensure consistency, accuracy, and reproducibility of the data.

### 2.10 TdT-Mediated dUTP Nick End Labeling (TUNEL) Staining

A2780 cells were divided into five experimental groups: negative control (NC), cisplatin (5 μg/mL), sh-RPS6KA2 + cisplatin, miR-512-3p mimics + cisplatin, and miR-512-3p mimics + RPS6KA2 + cisplatin. Similarly, A2780CP cells were classified into five groups: NC, cisplatin (20 μg/mL), RPS6KA2 + cisplatin, miR-512-3p inhibitor + cisplatin, and miR-512-3p inhibitor + sh-RPS6KA2 + cisplatin. Prior to processing, samples were brought to room temperature to prevent condensation upon opening. Tissue slides were rehydrated through immersion in phosphate-buffered saline (PBS, 0.01M, PH 7.4). DNA fragmentation was evaluated using the DeadEnd™ Fluorometric TUNEL System (Promega, Madison, WI, USA) following the manufacturer’s recommended protocol [28]. Briefly, specimens were fixed with 4% paraformaldehyde in PBS for 25 min at 4°C, washed twice with PBS, and permeabilization with 0.2% Triton X-100 in PBS for 5 min. After two additional washes with PBS, tissues were incubated in equilibration buffer for 10 min. The labeling reaction mixture was prepared as specified by the supplier. Samples were then incubated with the TUNEL reaction solution in a dark, humidified chamber at 37°C for 60 min. The enzymatic reaction was terminated by immersing the slides in 2× saline-sodium citrate for 15 min. Following three washes with PBS, nuclei were counterstained with DAPI (0.1 µg/mL in PBS) for 10 min and mounted using Mowiol® (Calbiochem, Darmstadt, Germany), supplemented with the antifade reagent 1,4-diazabicyclo [2.2.2] octane (Sigma, St. Louis, MO, USA). All procedures were independently repeated three times to ensure data reliability and reproducibility.

### 2.11 Autophagy Flux Assay

To evaluate autophagic activity, we observed the generation of fluorescent autophagosome puncta in A2780 and A2780CP cells transfected with an mRFP-GFP-LC3-tagged adenovirus (Hanbio Biotechnology Co., Shanghai, China). Cells were plated at a density of 2 × 10^5^ cells per well in 24-well plates and allowed to adhere overnight. After incubating with the adenovirus (diluted in serum-free medium) for 2 h, the inoculum was replaced with fresh medium, and cells were further cultured for 48 h. The cells were then rinsed twice with ice-cold PBS (0.01 M, PH 7.4), fixed, and counterstained with DAPI. Autophagy dynamics were examined using a high-content imaging system (Operetta CLS, Revvity, Waltham, MA, USA). Due to the acid-sensitive nature of GFP, fluorescence from the GFP-LC3 signal is quenched upon lysosomal fusion, while the mRFP signal remains stable, resulting in persistent red fluorescence. By overlaying green and red fluorescent signals, yellow puncta indicate autophagosomes prior to lysosomal fusion, whereas red-only puncta represent autolysosomes. For quantitative analysis, the number of yellow (autophagosomes) and red-only puncta (autolysosomes) per cell were recorded [29]. All assays were independently repeated three times to ensure experimental consistency and reproducibility.

### 2.12 Analysis of Mitochondrial Membrane Potential

A2780CP cells were washed gently with PBS (0.01 M, PH 7.4) and subsequently stained with BD™ MitoScreen JC-1 (BD Pharmingen, Franklin Lakes, NJ, USA) following the manufacturer’s protocol. After staining, cells were washed to remove excess dye and resuspended in MitoScreen buffer. Flow cytometric analysis was performed using a FACSCalibur flow cytometer (Becton-Dickinson, San Jose, CA, USA), where 30,000 events per sample were collected after daily calibration using BD CaliBRITE beads to ensure instrument consistency. The mitochondrial membrane potential was assessed by detecting red-fluorescent from JC-1 aggregates on the FL-2 channel and green fluorescence from monomeric JC-1 through the FL-1 channel [30]. Data processing and analysis were carried out using FCS Express 5 software (*De Novo* Software, Los Angeles, CA, USA). To confirm reproducibility and reliability, all experiments were independently repeated three times.

### 2.13 Single-Cell Sequencing Data Analysis

The relevant single-cell datasets, along with their annotated cell type information, were retrieved from the TISCH database (http://tisch.comp-genomics.org) in .h5 format [31]. Data processing and downstream analysis were carried out using the R packages ‘MAESTRO’ (version 0.6.3) and ‘Seurat’ (version 5.3.0). Cell clustering was reperformed using the t-SNE algorithm for improved visualization of cellular heterogeneity.

### 2.14 Glutathione (GSH) Analysis

GSH levels were measured using a GSH assay kit (KGT006, Jiangsu Kaiji Biotechnology Co., Ltd., Nanjing, China) following the manufacturer’s protocol. Briefly, 0.5 mL of each sample was mixed vigorously with 2 mL of the working solution. The mixture was then centrifuged at 3500–4000 rpm for 10 min using a Hitachi CP100NX centrifuge (Hitachi, Tokyo, Japan). After centrifugation, 1 mL of the supernatant was transferred and mixed with the assay reagent, followed by incubation at room temperature for 5 min. The absorbance was measured spectrophotometrically at 420 nm with a 1 cm pathlength cuvette, and the OD values were recorded and compared across samples. To ensure the consistency and reproducibility of the results, all assays were performed independently in triplicate.

### 2.15 Reactive Oxygen Species (ROS) Analysis

A2780CP were plated in 24-well plates at a density of 1 × 10^5^ cells per well and cultured overnight under standard conditions to ensure proper attachment. The following day, cells were loaded with fluorescent probes: 2^′^,7^′^-dichlorodihydrofluorescein diacetate (DCFH-DA, 10 μM) (Sigma-Aldrich, Darmstadt, Germany), dihydroethidium (DHE, 10 μM) (Sigma-Aldrich, Darmstadt, Germany), and 4-amino-5-methylamino-2^′^,7^′^-difluorofluorescein diacetate (DAF-FM DA, 5 μM) (Sigma-Aldrich, Darmstadt, Germany). After incubation for 30-min to allow probe uptake, excess dyes were removed by two washes with PBS (0.01 M, PH 7.4). The nuclei were counterstained with DAPI for 20 min at room temperature. Fluorescence intensity was measured using flow cytometry (ACEA Novocyte™ system, ACEA Biosciences, Inc., San Diego, CA, USA), with data collected from 10,000 gated events per sample. In parallel, fluorescent microscopy was performed to visualize intracellular signals using FluoView™ FV3000 (Thermo Fisher, Waltham, MA, USA). All experiments were independently repeated three times to ensure consistency and reproducibility of the findings.

### 2.16 Iron Measurement

Total iron content (Fe^2+^ and Fe^3+^) was assessed using the Iron Determination Kit (A039-1-1, Jiancheng Biotechnology Co., Ltd., Nanjing, China) following the manufacturer’s protocol. Briefly, samples were combined with the iron chromogenic reagent and heated in a boiling water bath for 5 min. After cooling to room temperature, the mixtures were centrifuged at 3500 revolutions per minute (RPM) for 10 min. A 1.0 mL portion of the resulting supernatant was then transferred to a cuvette, and absorbance was measured at 520 nm using an enzyme-linked immunosorbent assay (ELISA) reader (SPARK Cyto, Tecan SPARK, Männedorf, Switzerland). For detection of intracellular ferrous iron (Fe^2+^), the FerroOrange fluorescent probe (Catalog No. F374, Dojindo, Kumamoto, Japan) was employed according to the supplier’s instructions. All experiments were independently repeated three times to ensure experimental accuracy and reproducibility.

### 2.17 Clinical Specimen Collection

Between 01 January 2024, and 31 December 2024, three independent cohorts of ovarian cancer tissue and corresponding adjacent non-cancerous tissues were collected. Written informed consent was obtained from all participants prior to sample collection, and the study protocol was reviewed and approved by the Institutional Review Board of Zhoupu Hospital (Approval Number: 2025-C-209-E01). All experimental procedures were conducted in compliance with the ethical principles outlined in the 1995 Declaration of Helsinki.

### 2.18 Half Maximal Inhibitory Concentration (IC_***50***_) Analysis of Ovarian Cancer Tissues

Ovarian cancer tissues samples were obtained from clinical specimens and dissociated into single-cell suspensions for *in vitro* culture. Cells were seeded at a density of per well in 96-well plates and exposed to varying concentrations of drugs for 48 h. Cell viability was assessed using the CCK-8 assay according to the manufacturer’s instructions. Dose-response curves were generated by nonlinear regression analysis to determine the IC_50_. Each experiment was performed in triplicate, and the mean values were used for final analysis.

### 2.19 Expression Analysis of RPS6KA2 in Different Passages of Ovarian Cancer Cells

The expression of RPS6KA2 in mouse ovarian surface epithelial (MOSE) cells across different passage stages—early (passages 5–20), intermediate (passages 60–80), and late (passages 120–180)—were evaluated using whole genome microarray analyses from the gene expression dataset GDS3894 (https://www.ncbi.nlm.nih.gov/sites/GDSbrowser?acc=GDS3894 (accessed on 01 November 2025)) [32]. These cells underwent spontaneous transformation during prolonged culture, progressively evolving from non-tumorigenic state to intermediate and ultimately to aggressive, malignant phenotypes.

### 2.20 Expression Analysis of RPS6KA2 in Different Platinum-Based Drug-Sensitive Ovarian Cancer Tissues

Gene expression data for RPS6KA2 in ovarian cancer tissues with varying responses to carboplatin were retrieved from the publicly available dataset GDS1381 (https://www.ncbi.nlm.nih.gov/sites/GDSbrowser?acc=GDS1381 (accessed on 01 November 2025)) [33]. Primary cell cultures were derived from tumor specimens of six ovarian carcinoma patients and functionally characterized using the ChemoFx assay, which categorized them as either carboplatin-sensitive (n = 3) or carboplatin-resistant (n = 3). Genome-wide expression profiling was performed using Affymetrix U95A human gene chip arrays, with three replicates per culture (total n = 18). The analysis centered on assessing differences in RPS6KA2 expression levels between sensitive and resistant groups following carboplatintreatment for 2 or 72 h.

### 2.21 Analysis of RPS6KA2 Interacting Proteins

The potential interaction partners of RPS6KA2 were predicted through the STRING database (https://cn.string-db.org), and a detailed analysis of its protein–protein interaction network was conducted to explore functional relationships and interaction dynamics with associated proteins.

### 2.22 Analysis of the Relationship between Ferroptosis-Related Genes and RPS6KA2

RNA-seq data in the form of STAR-counts and corresponding clinical information for 375 ovarian cancer patients were retrieved from the TCGA database (https://portal.gdc.cancer.gov). Gene expression levels were normalized by converting counts to TPM format and applying a log2(TPM + 1) transformation. Patients were divided into high-expression (upper quartile, n = 96) and low-expression (lower quartile, n = 92) groups based on RPS6KA2 expression levels. The expression profiles of ferroptosis-related genes were then compared between these two groups. All statistical analyses were conducted using R software (version 4.0.3), and a *p* value < 0.05 was considered statistically significant.

### 2.23 Prediction of Potential Regulatory miRNAs of RPS6KA2

The potential microRNAs (miRNAs) regulating the RPS6KA2 gene were predicted using four publicly available databases: TargetScan (http://www.targetscan.org/), miRDB (http://www.mirdb.org/), miRWalk (http://mirwalk.umm.uni-heidelberg.de/ (accessed on 01 November 2025)), and StarBase (http://starbase.sysu.edu.cn/). Consensus miRNAs identified in all four platforms were extracted using Venny software (Version 2.1) to generate an intersection set, thereby enhancing the accuracy and reliability of the predicted regulatory miRNAs.

### 2.24 Immunohistochemical Analysis (IHC)

Tumor tissue samples were collected from clinical patients or established subcutaneous xenograft models. The mouse models were randomly divided into five groups (n = 6 per group) using a computer-generated randomization sequence. The first set included: normal control (NC), cisplatin treatment (Cis), sh-RPS6KA2 + Cis, miR-512-3p mimics + Cis, and miR-512-3p mimics + Cis + sh-RPS6KA2. The second set consisted of: NC, Cis, RPS6KA2 + Cis, miR-512-3p inhibitor + Cis, and miR-512-3p inhibitor + Cis + RPS6KA2. Following fixation in formaldehyde, immunohistochemical (IHC) analysis was performed to evaluate protein expression levels using primary antibodies against RPS6KA2 (14446-1-AP, 1:100 dilution; Proteintech, Chicago, USA), ATG5 (66744-1-Ig, 1:200 dilution; Proteintech, Chicago, USA), ATG7 (67341-1-Ig, 1:1000 dilution; Proteintech, Chicago, USA), BECN1 (66665-1-Ig, 1:500 dilution; Proteintech, Chicago, USA), SQSTM1 (66184-1-Ig, 1:3000 dilution; Proteintech, Chicago, USA). All stained tissue sections were independently assessed by a qualified pathologist blinded to the experimental conditions.

### 2.25 Artificial Intelligence (AI) Tools

In this study, the artificial intelligence tool DeepSeek-R1 (Hangzhou DeepSeek AI Foundation Technology Research Co., Ltd., Hangzhou, China) was employed to polish the language of the article and improve its readability.

### 2.26 Statistics Analysis

Statistical analysis of the collected data was conducted using SPSS 22.0 software (IBM, Armonk, NY, USA), employing a range of methods such as *t*-tests, paired *t*-tests, one-way analysis of variance (ANOVA), and Wilcoxon tests, depending on the data type and distribution. For data following a normal distribution, results were expressed as mean ± standard deviation. Categorical variables were analyzed using the chi-square test, chi-square test with continuity correction, or Fisher’s exact test, as appropriate. The Wilcoxon rank-sum test was utilized to assess associations between RPS6KA2 expression levels and tumor proliferation, apoptosis, or the PI3K-AKT-mTOR signaling pathway. All graphical representations were created using GraphPad Prism 9.0 (Dotmatics, San Diego, CA, USA). A *p* value below 0.05 was considered indicative of statistical significance.

## 3 Result

### 3.1 Relationship between RPS6KA2 and the Occurrence, Progression and Prognosis of Ovarian Cancer

**Figure 1:**
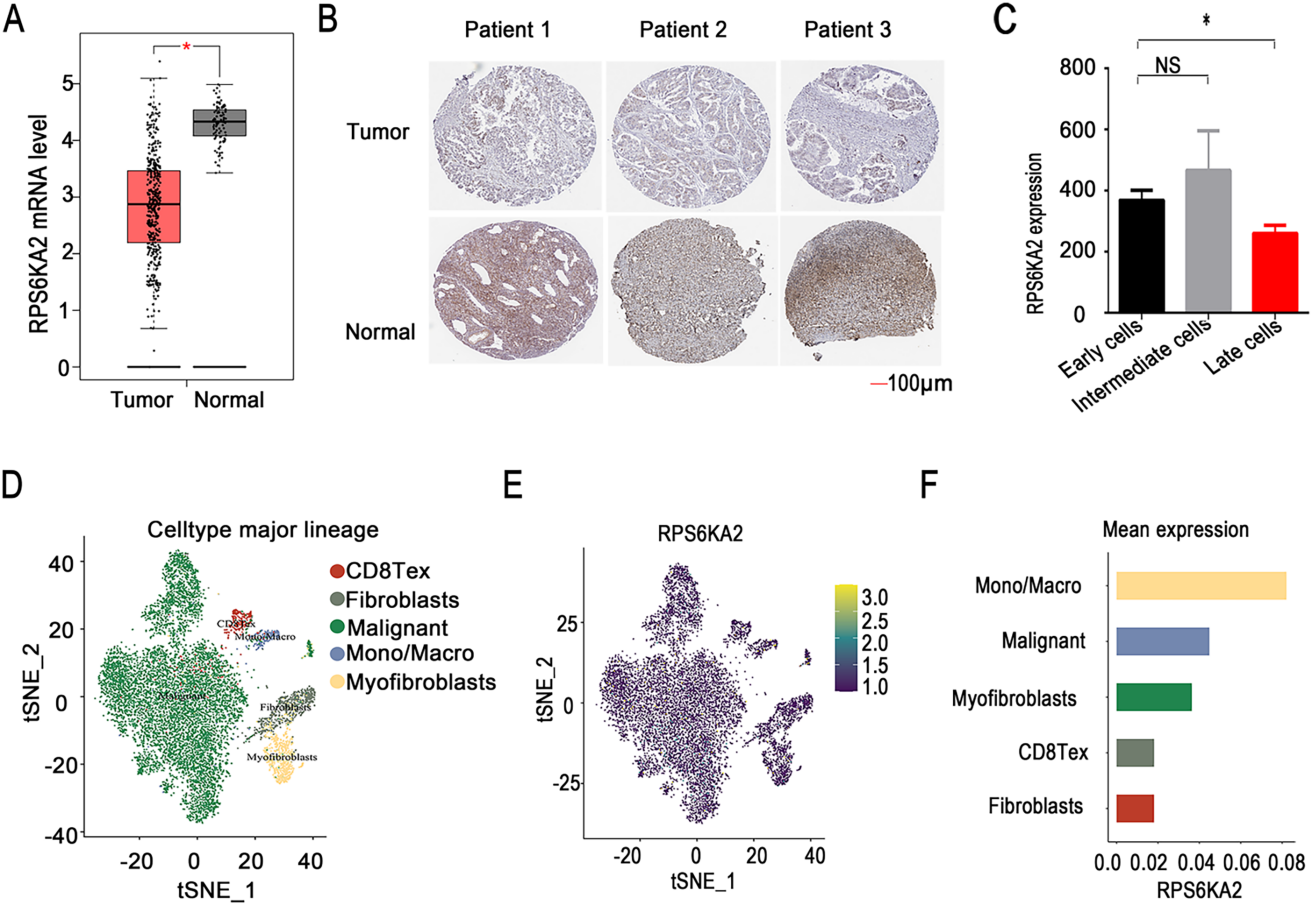

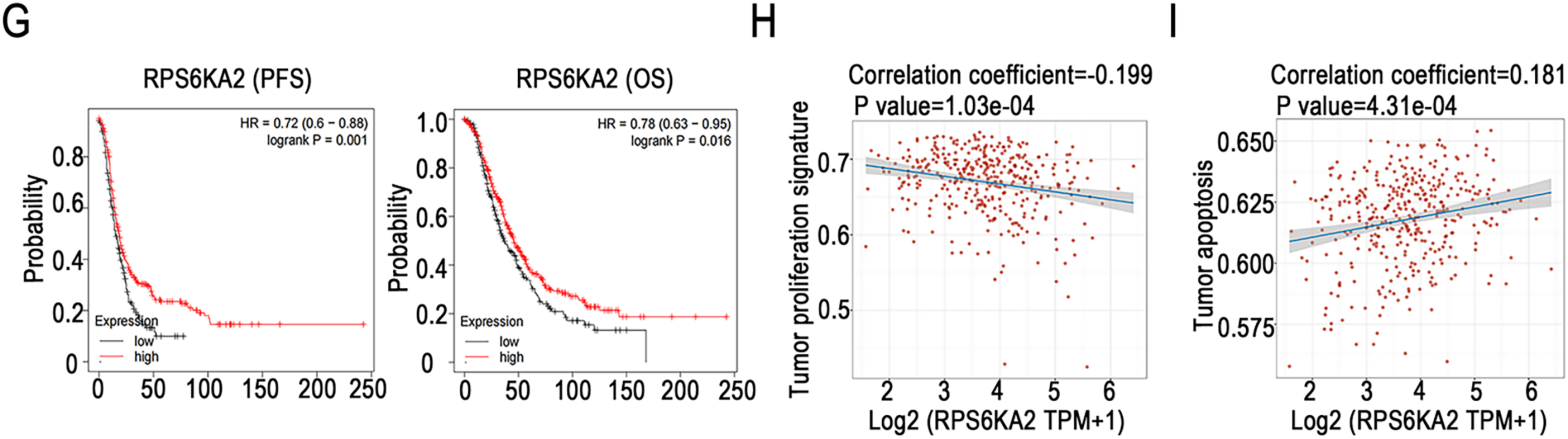
RPS6KA2's role in ovarian cancer initiation, progression, prognosis, cell proliferation, and apoptosis. (**A**) Comparison of RPS6KA2 expression levels between ovarian tumor tissues (n = 426) and normal tissues (n = 88) using the GEPIA database; (**B**) immunohistochemical staining showing RPS6KA2 expression in ovarian tumor tissues (n = 3) and adjacent normal tissues (n = 3); (**C**) expression of RPS6KA2 expression across early (passages 5–20), intermediate (passages 60–80), and late (passages 120–180) passage mouse ovarian surface epithelial cells (MOSE); (**D**) t-SNE plot depicting single-cell clustering, with distinct colors representing different cell populations; (**E**) t-SNE map illustrating the spatial distribution of RPS6KA2 expression levels across individual cells, where color intensity reflects expression magnitude; (**F**) bar graph summarizing RPS6KA2 expression abundance across different cell types; (**G**) survival analysis based on the Kaplan-Meier Plotter database, evaluating the relationship of RPS6KA2 expression with progression-free survival (PFS) and overall survival (OS). Correlation analysis between RPS6KA2 mRNA levels and tumor proliferation (**H**) or apoptosis (**I**) pathway scores, with the *x*-axis representing RPS6KA2 expression distribution and the *y*-axis indicating pathway activity scores. *p < 0.05; NS, not significant (*p* > 0.05)

RPS6KA2 expression levels were markedly reduced in ovarian tumor tissues relative to normal controls (Fig. 1A,B), implying a potential role in the initiation of ovarian cancer. This downregulation was further accentuated in mouse ovarian surface epithelial cells with increasing passage number (Fig. 1C), suggesting its possible involvement in cancer progression. Single-cell sequencing data showed that among various cell types in ovarian cancer tissues, monocytes and macrophages exhibited the highest RPS6KA2 expression, tumor cells displayed moderate levels, while fibrocytes had the lowest expression (Fig. 1D–F). Notably, Kaplan-Meier survival analysis showed that patients with low RPS6KA2 expression experienced significantly poorer progression-free and overall survival rates (Fig. 1G), underscoring its potential as a prognostic biomarker. Furthermore, this study investigated the relationship between RPS6KA2 expression and key signaling pathways associated with cell proliferation (Fig. 1H) and apoptosis (Fig. 1I) in ovarian cancer. The results indicated that RPS6KA2 levels are inversely correlated with tumor cell proliferation and positively associated with apoptotic activity, supporting the hypothesis that RPS6KA2 may function to suppress tumor growth by inhibiting proliferation and enhancing apoptosis.

### 3.2 Relationship between RPS6KA2 and Cisplatin Resistance in Ovarian Cancer

**Figure 2:**
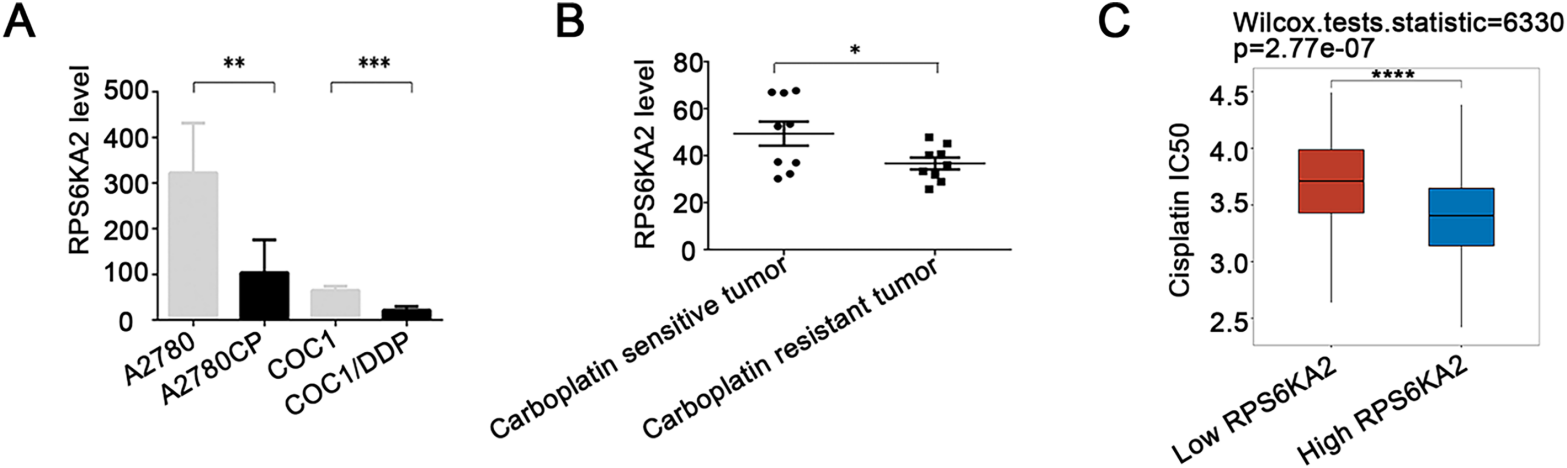

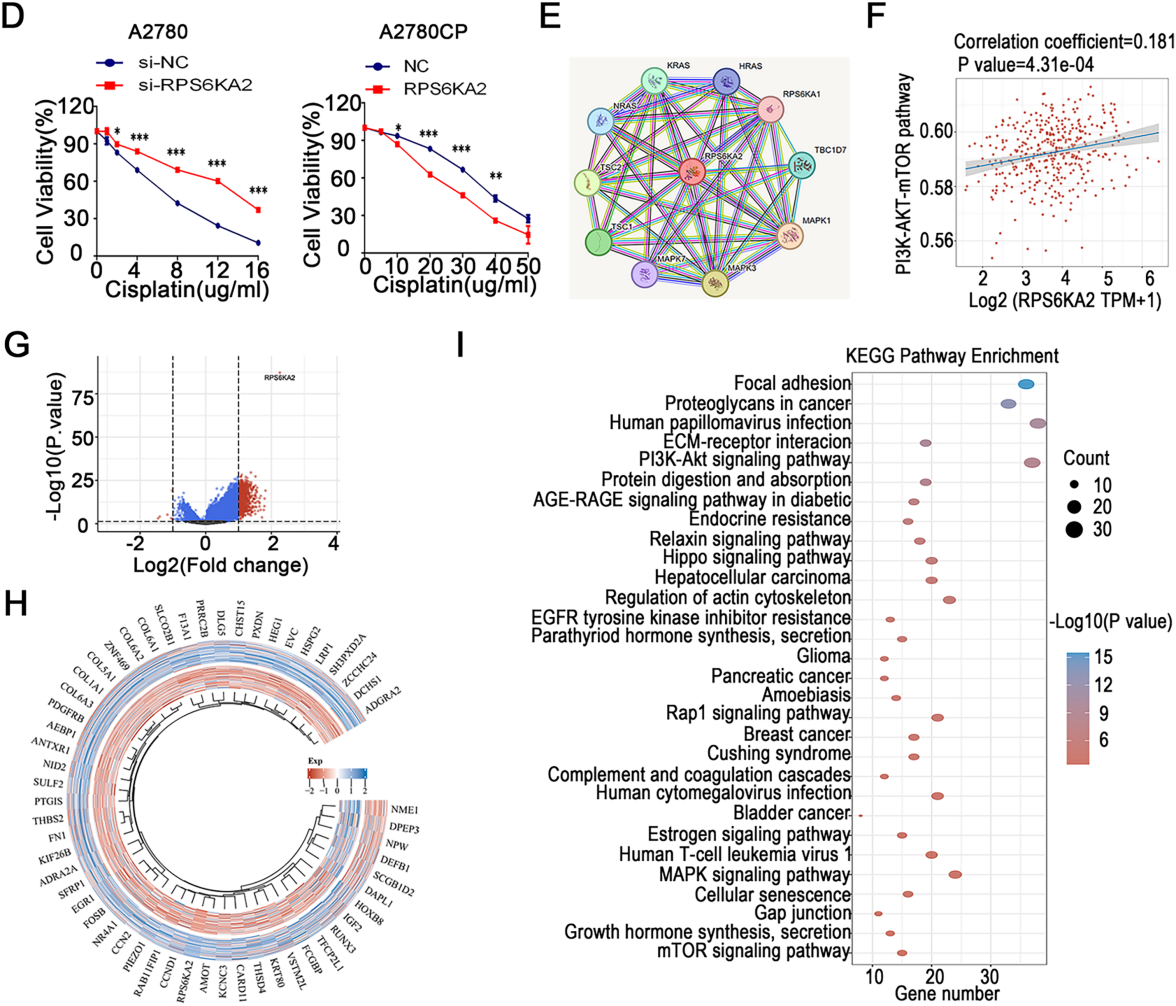
The association between RPS6KA2 and cisplatin resistance, along with the identification of its downstream target genes. (**A**) RPS6KA2 mRNA expression levels were compared between cisplatin-resistant (A2780CP and COC1/DDP) and cisplatin-sensitive cell lines (A2780 and COC1); (**B**) RPS6KA2 mRNA expression was evaluated in ovarian cancer tissues from cisplatin-resistant (n = 9) and cisplatin-sensitive (n = 9) patients; (**C**) Wilcoxon rank-sum test was used to assess the correlation between cisplatin IC_50_ values and RPS6KA2 expression levels. The *x*-axis represents different sample groups, and the *y*-axis shows the distribution of IC_50_ scores; (**D**) a CCK-8 assay was performed to evaluate cell viability following treatment with increasing concentrations of cisplatin, after either silencing RPS6KA2 in A2780 cells or overexpressing it in A2780CP cells; (**E**) protein–protein interaction networks involving RPS6KA2 were analyzed using the STRING database; (**F**) the correlation between PI3K-AKT-mTOR pathway activity scores and RPS6KA2 expression levels was examined. The *x*-axis displays the distribution of RPS6KA2 expression, and the *y*-axis reflects the distribution of pathway activity score; (**G**) among 188 ovarian cancer patients, stratification into low (n = 92) and high (n = 96) RPS6KA2 expression groups was conducted. Each dot represents an individual gene, with colors indicating whether predefined filtering criteria were met; (**H**) a heatmap was constructed to visualize the expression patterns of differentially expressed genes, with sample groups ordered from the outermost to the innermost layer; (**I**) KEGG pathway enrichment analysis was conducted, where color intensity indicates the level of statistical significance and circle size corresponds to the number of enriched genes (larger circles indicate more enriched genes). *p < 0.05; **p < 0.01; ***p < 0.001; ****p < 0.0001

The results showed that RPS6KA2 expression was markedly decreased in platinum-resistant cells and tissues when compared to platinum-sensitive samples (Fig. 2A,B), indicating a possible link between downregulation of RPS6KA2 and the development of cisplatin resistance. Moreover, patients with low RPS6KA2 expression were found to have higher IC_50_ values for cisplatin than those with high expression (Fig. 2C), reinforcing the association between reduced RPS6KA2 and diminished drug sensitivity. Consistent with this, CCK8 analysis demonstrated that, upon exposure to different concentrations of cisplatin, ovarian cancer cells with low RPS6KA2 expression displayed enhanced cell viability, whereas those with high RPS6KA2 expression showed greater sensitivity and reduced survival (Fig. 2D). Collectively, these findings strongly support the role of low RPS6KA2 expression in promoting cisplatin resistance in ovarian cancer.

### 3.3 RPS6KA2 Modulates Cisplatin Sensitivity in Ovarian Cancer through the mTOR Signaling Pathway

Analysis using the STRING database (https://cn.string-db.org) revealed that RPS6KA2 physically interacts with key autophagy regulators, TSC complex subunit 1 (TSC1) and TSC complex subunit 2 (TSC2), and is positively associated with the phosphatidylinositol 3-kinase-protein kinase B-mammalian target of rapamycin (PI3K-mTOR) signaling cascade (Fig. 2E,F). A high-throughput sequencing comparison between 92 ovarian cancer patients with low RPS6KA2 expression and 96 with high expression identified 617 upregulated and 6 downregulated genes in the high-expression cohort (Fig. 2G,H). KEGG pathway enrichment analysis indicated that these differentially expressed genes are significantly enriched in multiple signaling pathways, particularly the mTOR pathway (Fig. 2I), implying a potential role for RPS6KA2 in modulating cisplatin sensitivity via regulation of autophagy.

**Figure 3:**
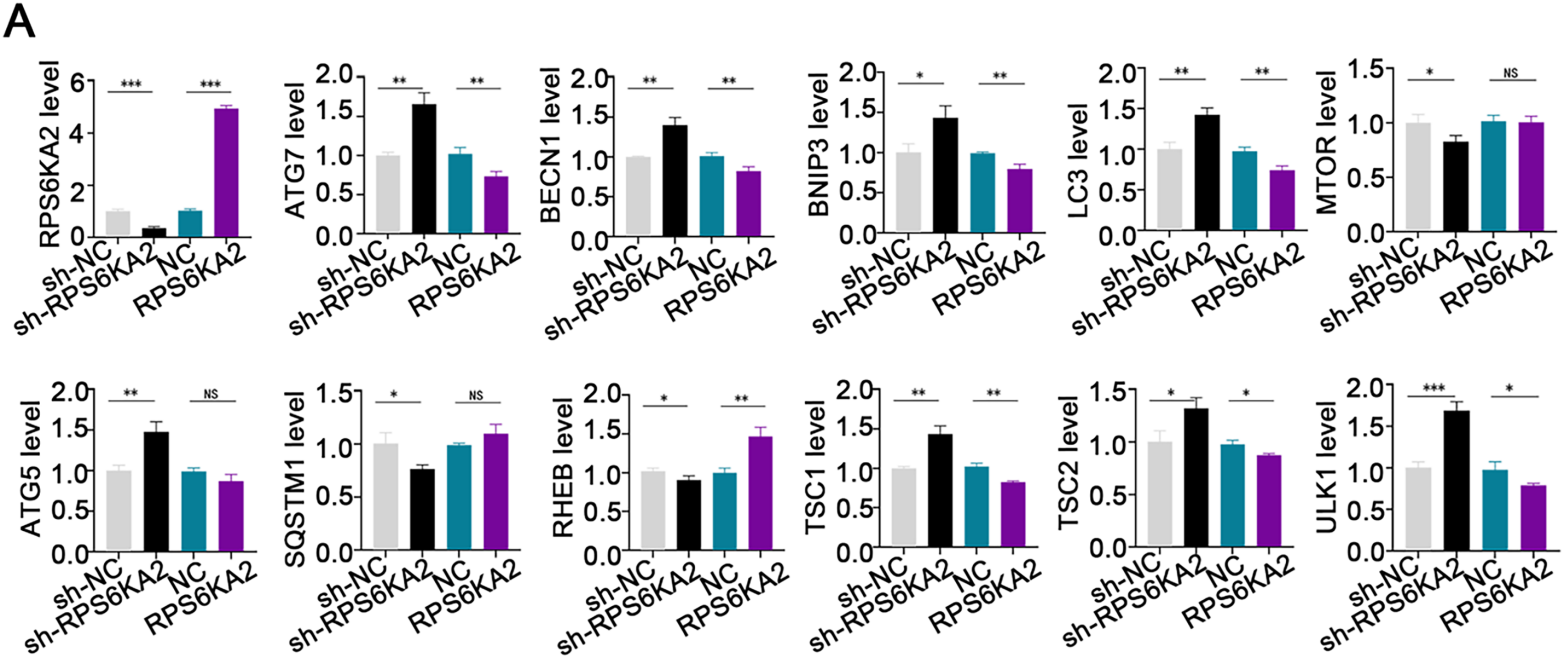

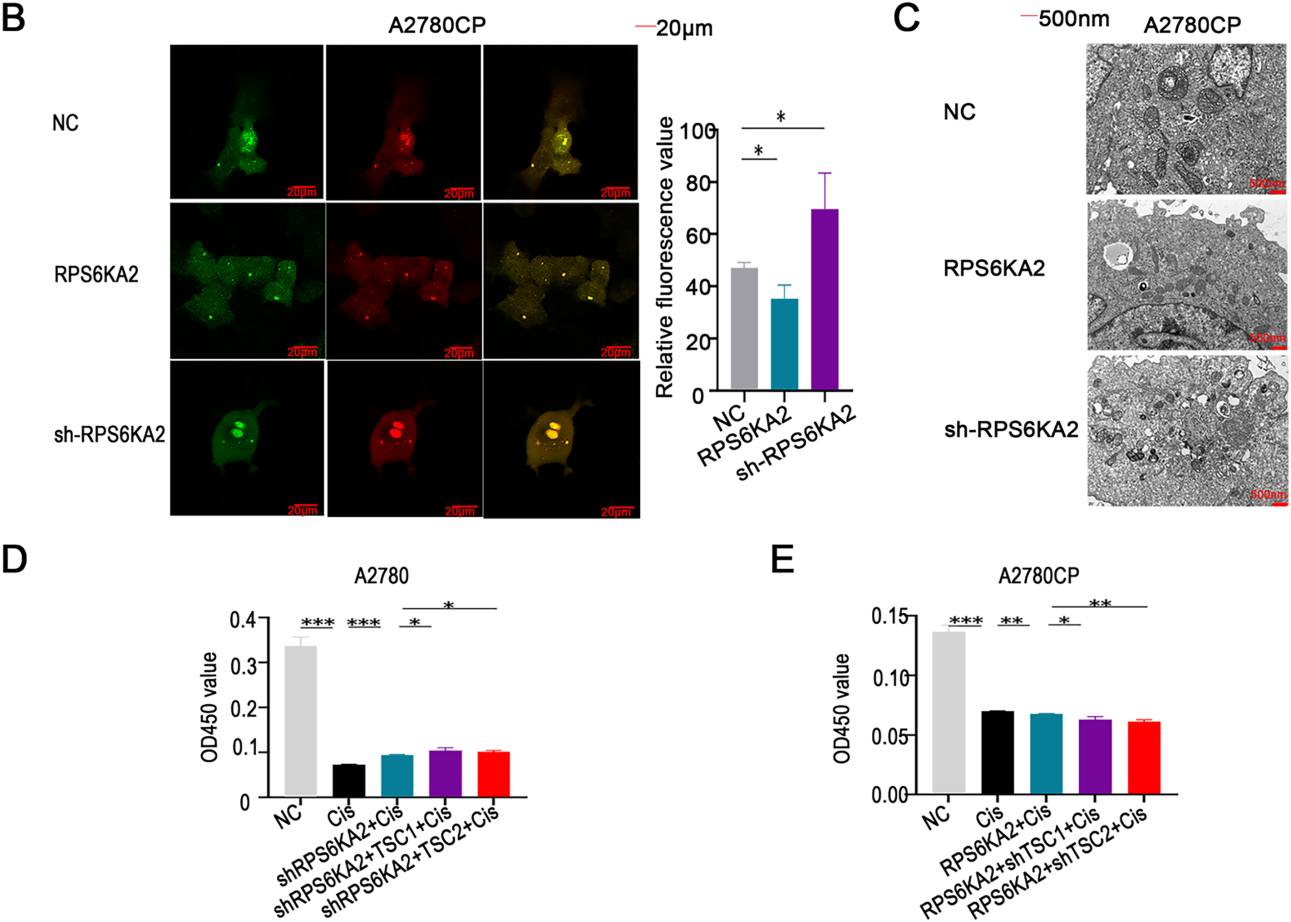
Involvement of RPS6KA2 in the regulation of autophagy and cisplatin sensitivity in ovarian cancer cells. (**A**) qRT-PCR analysis was conducted to measure RPS6KA2 and autophagy-related proteins in ovarian cancer cell lines following transfection with either an RPS6KA2 overexpression plasmid or sh-RPS6KA2; (**B**) autophagic flux in A2780CP cells was evaluated using mRFP-GFP-LC3 fluorescence assay after transfection with RPS6KA2 plasmid or sh-RPS6KA2. Green signals correspond to autophagosomes, red signals indicate autolysosomes, and yellow signals reflect the early stage of autophagosome formation; (**C**) high-resolution transmission electron microscopy (×10,000 magnification) was utilized to observe ultrastructural changes associated with autophagosome formation in A2780CP cells post-transfection; (**D**) A2780 cells were categorized into five experimental groups: NC, Cisplatin (5 μg/mL), sh-RPS6KA2 + Cisplatin, sh-RPS6KA2 + TSC1 + Cisplatin, and sh-RPS6KA2 + TSC2 + Cisplatin. Cell viability was determined by CCK-8 assay (OD_450_ values). (**E**) A2780CP cells were allocated into five groups: NC, Cisplatin (20 μg/mL), RPS6KA2 + Cisplatin, RPS6KA2 + sh-TSC1 + Cisplatin, and RPS6KA2 + sh-TSC2 + Cisplatin. Changes in OD_450_ values were measured using the CCK-8 assay. *p < 0.05; **p < 0.01; ***p < 0.001; NS, not significant (*p* > 0.05)

Suppression of RPS6KA2 led to increased expression of key autophagy-related genes, such as autophagy related 7 (ATG7), beclin 1 (BECN1), BCL2 interacting protein 3 (BNIP3), microtubule associated protein 1 light chain 3 alpha (LC3), autophagy related 5 (ATG5), TSC1, TSC2, and unc-51 like autophagy activating kinase 1 (ULK1), while reducing levels of mechanistic target of rapamycin kinase (MTOR), sequestosome 1 (SQSTM1), and Ras homolog, mTORC1 binding (RHEB) (Fig. 3A). In contrast, overexpression of RPS6KA2 resulted in decreased expression of these autophagy-related genes and elevated RHEB levels (Fig. 3A). Functional validation using mRFP-GFP-LC3 assays and transmission electron microscopy demonstrated that RPS6KA2 overexpression suppressed autophagic flux and autophagosome formation, whereas its downregulation promoted both processes (Fig. 3B,C).

Functional studies using CCK8 assays showed that silencing RPS6KA2 (sh-RPS6KA2) increased cell viability upon cisplatin treatment, as indicated by increased OD_450_ values, and this chemoresistant phenotype was further enhanced by co-overexpression of TSC1/TSC2 (Fig. 3D). In contrast, ectopic expression of RPS6KA2 sensitized cells to cisplatin, reflected by lower OD_450_ values, and the effect was intensified when TSC1/TSC2 were knocked down (Fig. 3E). These findings collectively suggest that RPS6KA2 suppresses autophagy through activation of the mTOR pathway, thereby counteracting cisplatin resistance in ovarian cancer.

### 3.4 RPS6KA2 Promotes Ferroptosis in Ovarian Cancer

**Figure 4:**
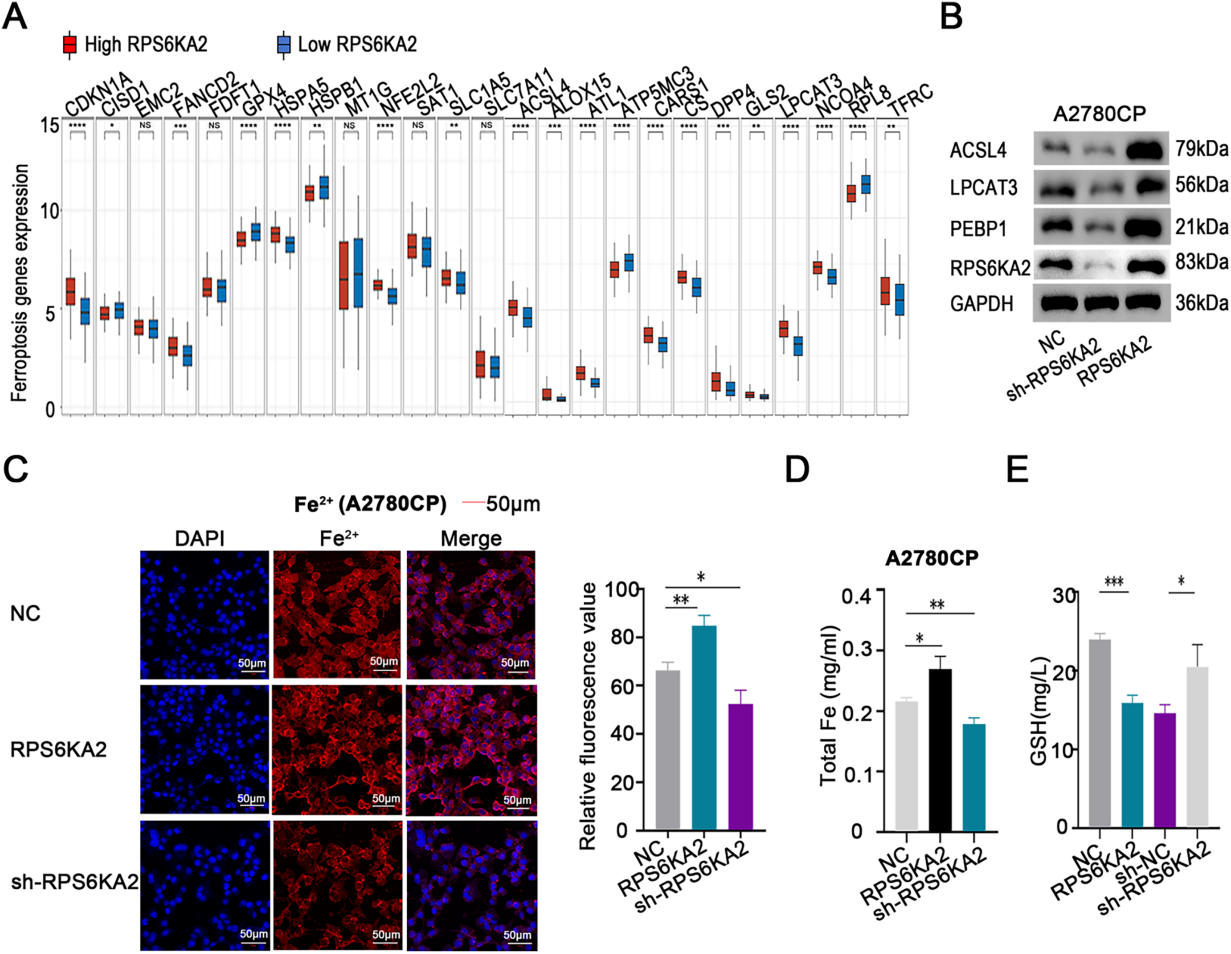

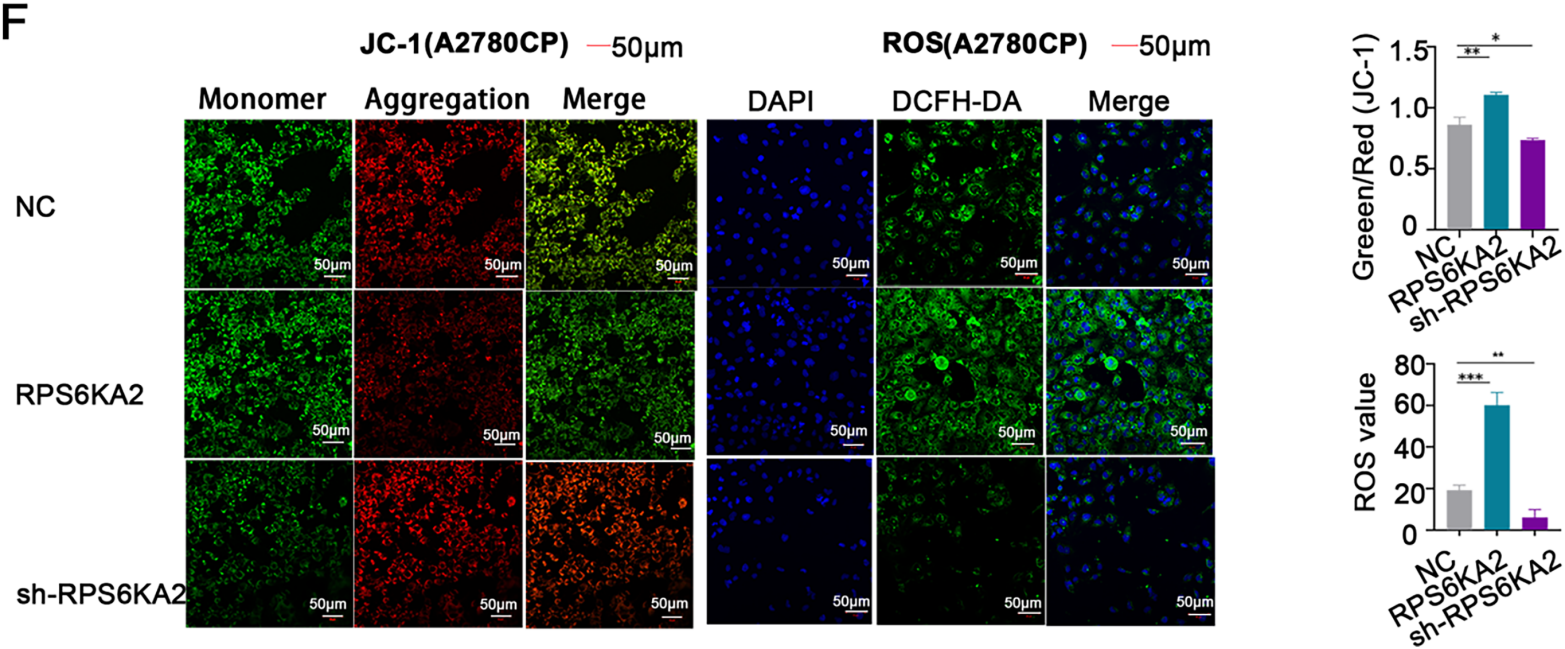
Involvement of RPS6KA2 in the regulation of ferroptosis in ovarian cancer cells. (**A**) Heatmaps display the expression patterns of ferroptosis-related genes in tumor and normal tissues. The *x*-axis corresponds to various ferroptosis-related genes, while the *y*-axis reflects their relative expression levels; (**B**) Western blotting was used to examine alterations in the expression of ferroptosis-related protein expression in A2780CP cells following transfection with either RPS6KA2 overexpression plasmid or sh-RPS6KA2; (**C**) levels of intracellular Fe^2+^ levels were detected by fluorescence staining (blue fluorescence marks nuclei; red fluorescence indicates Fe^2+^ presence); (**D**) total iron content was quantified in A2780CP cells under different treatment conditions; (**E**) cellular GSH levels were measured as an indicator of antioxidant capacity; (**F**) changes in mitochondrial membrane potential were assessed using fluorescent probes. Green fluorescence indicates high mitochondrial membrane potential (viable cells), while red fluorescence signifies low membrane potential (apoptotic cells); Intracellular ROS accumulation was evaluated using fluorescent probes. With blue fluorescence labeling nuclei, and green fluorescence also reflecting ROS accumulation. *p < 0.05; **p < 0.01; ***p < 0.001; ****p < 0.0001; NS, not significant (*p* > 0.05)

Evaluation of ferroptosis-linked gene expression profiles indicated that individuals with low RPS6KA2 levels displayed markedly diminished expression of the majority of genes implicated in ferroptosis (e.g., cyclin dependent kinase inhibitor 1A (CDKN1A), FA complementation group D2 (FANCD2), heat shock protein family A (Hsp70) member 5 (HSPA5), NFE2 like bZIP transcription factor 2 (NFE2L2), solute carrier family 1 member 5 (SLC1A5), acyl-CoA synthetase long chain family member 4 (ACSL4), arachidonate 15-lipoxygenase (ALOX15), atlastin GTPase 1 (ATL1), cysteinyl-tRNA synthetase 1 (CARS1), citrate synthase (CS), dipeptidyl peptidase 4 (DPP4), glutaminase 2 (GLS2), lysophosphatidylcholine acyltransferase 3 (LPCAT3), nuclear receptor coactivator 4 (NCOA4), and transferrin receptor (TFRC)). In contrast, in patients exhibiting high RPS6KA2 expression, a distinct set of genes were downregulated, including CDGSH iron sulfur domain 1 (CISD1), glutathione peroxidase 4 (GPX4), heat shock protein family B (small) member 1 (HSPB1), ribosomal protein L8 (RPL8), and ATP synthase membrane subunit c locus 3 (ATP5MC3) (Fig. 4A). These findings imply a potential regulatory function of RPS6KA2 in the ferroptotic pathway.

Follow-up studies showed that silencing RPS6KA2 led to decreased protein levels of key ferroptosis-related factors such as ACSL4, LPCAT3, and PEBP1, whereas enhancing RPS6KA2 expression resulted in their upregulation (Fig. 4B). Similarly, overexpression of RPS6KA2 led to the increase of intracellular Fe²^+^ and total iron concentrations, while suppression of it reduced these levels (Fig. 4C,D). Considering the recognized role of iron accumulation in driving ferroptosis, these results reinforce the notion that RPS6KA2 facilitates ferroptosis.

Moreover, elevated RPS6KA2 expression correlated with lower levels of GSH, a critical suppressor of ferroptosis, whereas its reduction was linked to higher GSH levels (Fig. 4E), providing additional evidence for its pro-ferroptotic function. Mitochondrial membrane potential assessments using JC-1 staining demonstrated that heightened RPS6KA2 expression augmented apoptotic signals, whereas its depletion diminished them (Fig. 4F), aligning with known mechanisms linking mitochondrial dysfunction to ferroptosis..

Additionally, increasing RPS6KA2 expression boosted intracellular ROS generation, whereas reducing its expression suppressed ROS accumulation (Fig. 4F). Since ROS production is a hallmark of ferroptosis progression, these findings collectively indicate that RPS6KA2 contributes to the induction and regulation of ferroptosis in ovarian cancer.

### 3.5 miR-512-3p Promotes Cisplatin Resistance in Ovarian Cancer by Inhibiting RPS6KA2 Expression

**Figure 5:**
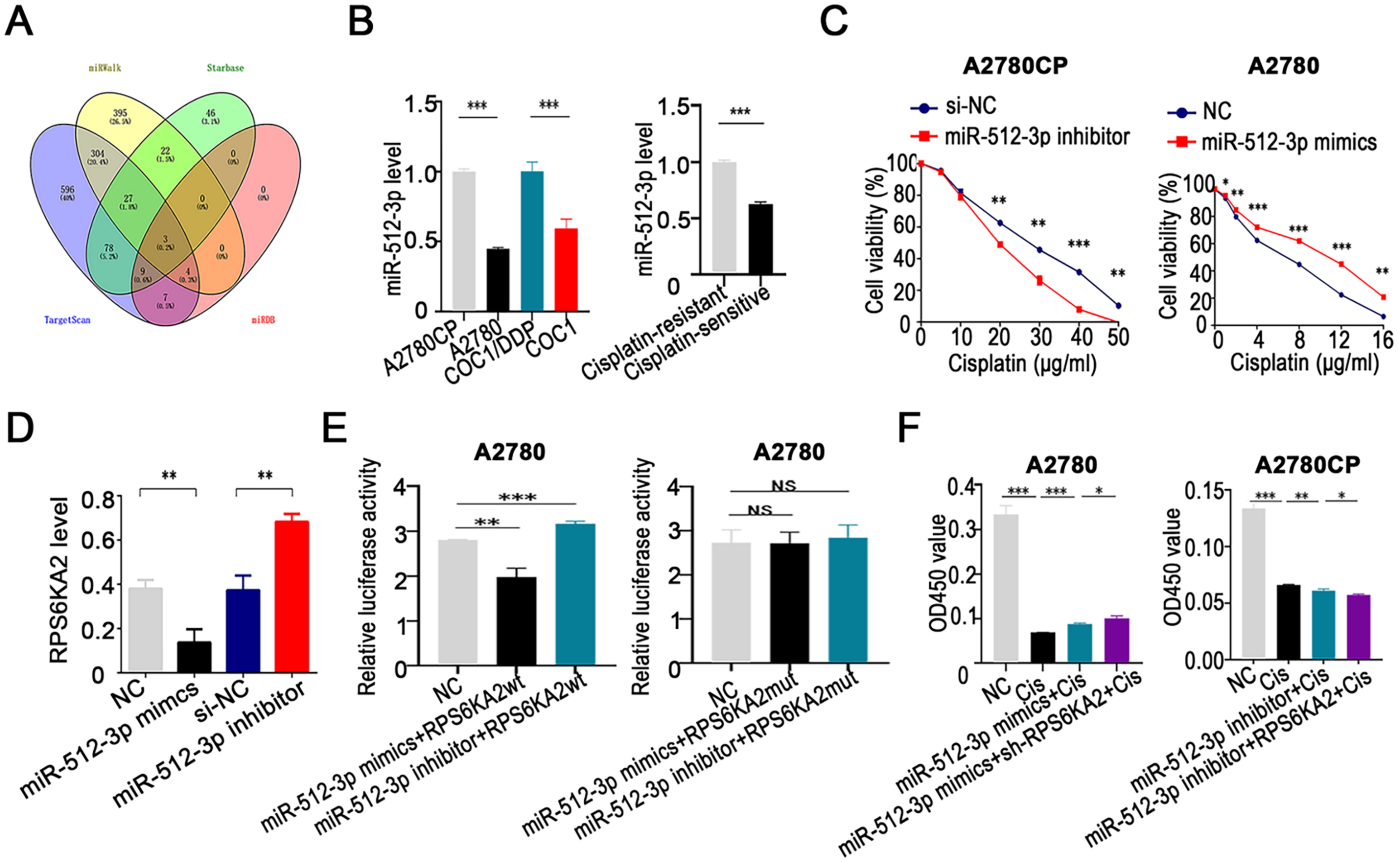

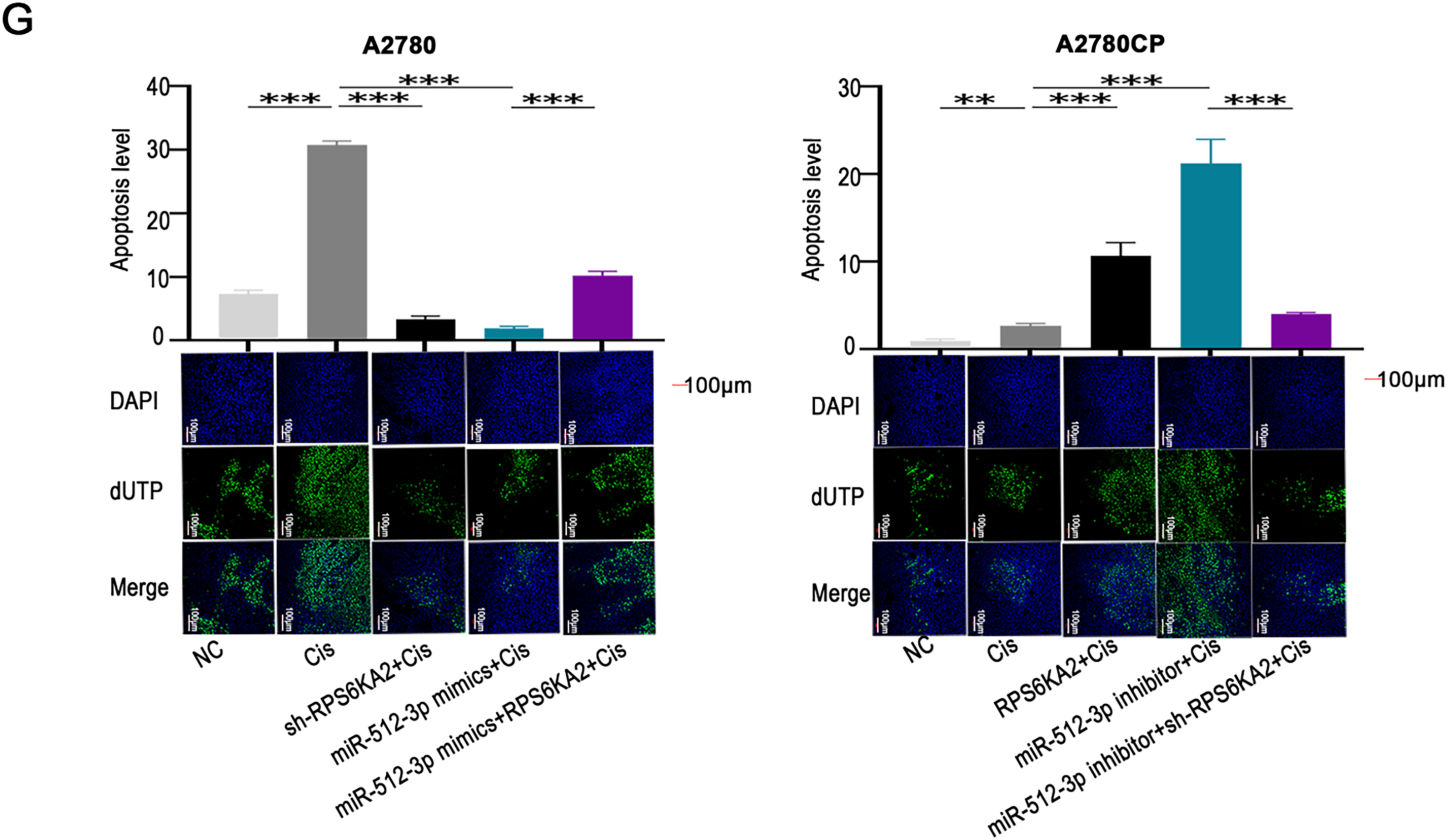
miR-512-3p enhances cisplatin resistance in ovarian cancer through direct targeting of RPS6KA2. (**A**) A comprehensive bioinformatics analysis was performed to identify upstream miRNAs that regulate RPS6KA2, using four public databases: TargetScan, miRDB, miRWalk, and StarBase databases; (**B**) expression levels of miR-512-3p were analyzed in cisplatin-resistant cell lines (A2780CP and COC1/DDP) vs cisplatin-sensitive counterparts (A2780 and COC1), as well as in clinical samples from cisplatin-resistant (n = 3) and cisplatin-sensitive (n = 3) ovarian cancer tissues; (**C**) cell viability following cisplatin exposure at different concentrations was evaluated using the CCK-8 assay after silencing miR-512-3p in A2780CP cells or overexpressing it in A2780 cells; (**D**) qRT-PCR was employed to determine alterations in RPS6KA2 expression upon transfection with either miR-512-3p mimics or inhibitor; (**E**) relative luciferase activity was measured in A2780 cells co-transfected with miR-512-3p mimics or inhibitor in combination with wild-type (wt) or mutant (mut) RPS6KA2 plasmids; (**F**) A2780 cells were grouped into four conditions: negative control (NC), cisplatin (5 μg/mL), miR-512-3p mimics + cisplatin, and miR-512-3p mimics + sh-RPS6KA2 + cisplatin. Similarly, A2780CP cells were divided into four groups: NC, cisplatin (20 μg/mL), miR-512-3p inhibitor + cisplatin, and miR-512-3p inhibitor + RPS6KA2 + cisplatin. Cell viability was assessed using the CCK-8 assay; (**G**) Apoptosis was examined by TUNEL staining in five experimental groups for each cell line. In A2780 cells: NC, cisplatin (5 μg/mL), sh-RPS6KA2 + cisplatin, miR-512-3p mimics + cisplatin, and miR-512-3p mimics + RPS6KA2 + cisplatin. In A2780CP cells: NC, cisplatin (20 μg/mL), RPS6KA2 + cisplatin, miR-512-3p inhibitor + cisplatin, and miR-512-3p inhibitor + sh-RPS6KA2 + cisplatin. Nuclei were stained blue, and apoptotic cells exhibited green fluorescence. *p < 0.05; **p < 0.01; ***p < 0.001; NS, not significant (*p* > 0.05)

**Figure A1:**
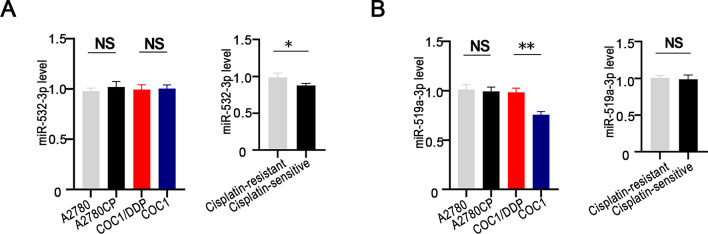
Expression of miR-532-3p and miR-519a-3p in ovarian cancer cells and tissues with different cisplatin sensitivity. miR-532-3p (**A**) or miR-519a-3p (**B**) expression levels were compared between cisplatin-resistant (A2780CP and COC1/DDP) and cisplatin-sensitive (A2780 and COC1) cell lines, as well as between cisplatin-resistant (n = 3) and cisplatin-sensitive (n = 3) ovarian cancer tissues. *p < 0.05; **p < 0.01; NS, not significant (*p* > 0.05)

To identify upstream miRNAs that regulate RPS6KA2, a comprehensive screening was conducted across four bioinformatics databases (TargetScan (http://www.targetscan.org/), miRDB (http://www.mirdb.org/), miRWalk (http://mirwalk.umm.uni-heidelberg.de/ (accessed on 01 November 2025)), and StarBase (http://starbase.sysu.edu.cn/)). This analysis yielded three candidate miRNAs: hsa-miR-532-3p, hsa-miR-519a-3p, and hsa-miR-512-3p (Fig. 5A). Subsequent validation in both cisplatin-sensitive and cisplatin-resistant ovarian cancer cell lines and tissues showed that miR-512-3p expression was markedly upregulated in resistant samples relative to sensitive ones (Figs. 5B and A1), implicating its potential role in mediating drug resistance.

Functional experiments indicated that silencing miR-512-3p enhanced the sensitivity of ovarian cancer cells to cisplatin, whereas its overexpression conferred greater resistance (Fig. 5C). Further investigation demonstrated that miR-512-3p significantly downregulated RPS6KA2 expression (Fig. 5D). A dual-luciferase reporter assay confirmed the direct interaction between miR-512-3p and the 3^′^ untranslated region (3^′^UTR) of RPS6KA2, leading to translational repression (Fig. 5E).

Notably, co-administration of sh-RPS6KA2, miR-512-3p mimics, and cisplatin resulted in higher cell viability (OD_450_) compared to cells treated with miR-512-3p mimics alone (Fig. 5F). Conversely, simultaneous treatment with RPS6KA2 overexpression vector, miR-512-3p inhibitors, and cisplatin led to a significant reduction in cell survival (Fig. 5F). These results indicate that miR-512-3p promotes cisplatin resistance by targeting and suppressing RPS6KA2.

Apoptosis assays revealed that either knockdown of RPS6KA2 or introduction of miR-512-3p mimics alone diminished cisplatin-induced apoptotic cell death (Fig. 5G). However, when RPS6KA2 was overexpressed alongside miR-512-3p mimics, apoptosis levels were restored (Fig. 5G). Similarly, individual treatments with RPS6KA2 overexpression or miR-512-3p inhibitors alone increased apoptosis, but this pro-apoptotic effect was abolished when miR-512-3p inhibitors were combined with sh-RPS6KA2 (Fig. 5G). Collectively, these findings suggest that miR-512-3p contributes to cisplatin resistance in ovarian cancer cells by inhibiting apoptosis through negative regulation of RPS6KA2 expression.

### 3.6 miR-512-3p Activates Autophagic Signaling through Inhibition of RPS6KA2 in Ovarian Cancer

**Figure 6:**
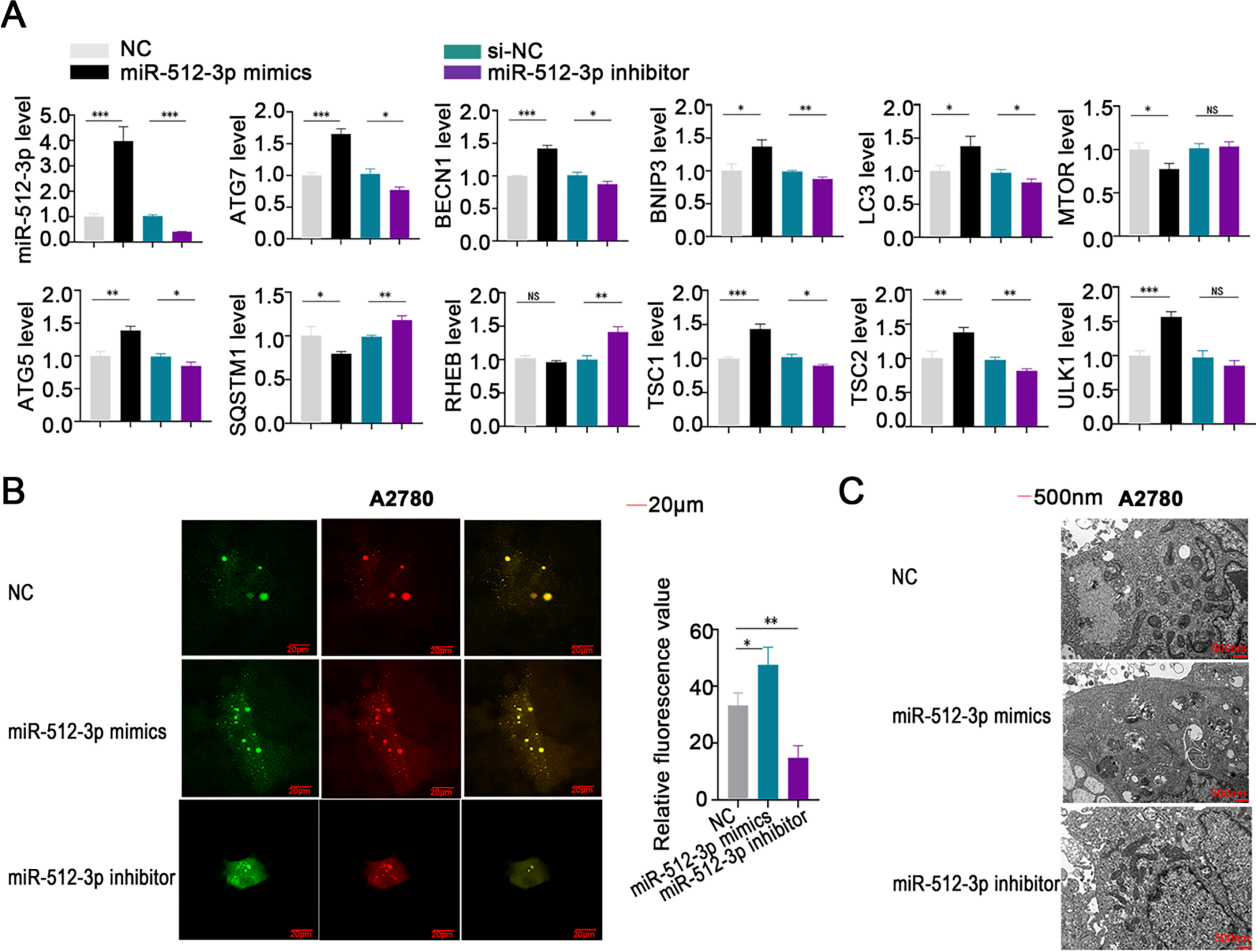
Involvement of miR-512-3p in the regulation of autophagy in ovarian cancer cells. (**A**) qRT-PCR analysis was conducted to assess alterations in miR-512-3p levels and expression of autophagy-related genes in ovarian cancer cell lines following transfection with miR-512-3p mimics or inhibitors; (**B**) autophagic flux in A2780 cells was evaluated using the mRFP-GFP-LC3 analysis after transfection. Green fluorescence indicates autophagosomes, red fluorescence indicates autolysosomes, and yellow puncta reflect the early formation stage of autophagosomes. (**C**) High-resolution transmission electron microscopy (×10,000 magnification) was applied to examine ultrastructural changes in autophagosome formation in transfected A2780 cells. *p < 0.05; **p < 0.01; ***p < 0.001; NS, not significant (*p* > 0.05)

Elevated expression of miR-512-3p led to a significant increase in the levels of autophagy-related genes, including ATG7, BECN1, BNIP3, LC3, ATG5, TSC1, TSC2, and ULK1, while simultaneously reducing the expression of MTOR and SQSTM1 (Fig. 6A). In contrast, suppression of miR-512-3p produced opposite effects, including elevated SQSTM1 and RHEB expression (Fig. 6A). Autophagic flux was further assessed using the mRFP-GFP-LC3 assay, which showed that overexpression of miR-512-3p enhanced autophagic flux, whereas its suppression attenuated flux activity (Fig. 6B). Transmission electron microscopy demonstrated a higher number of autophagosomes upon miR-512-3p overexpression and fewer autophagic structures upon its downregulation (Fig. 6C), reinforcing the role of miR-512-3p in promoting autophagy in ovarian cancer.

**Figure 7:**
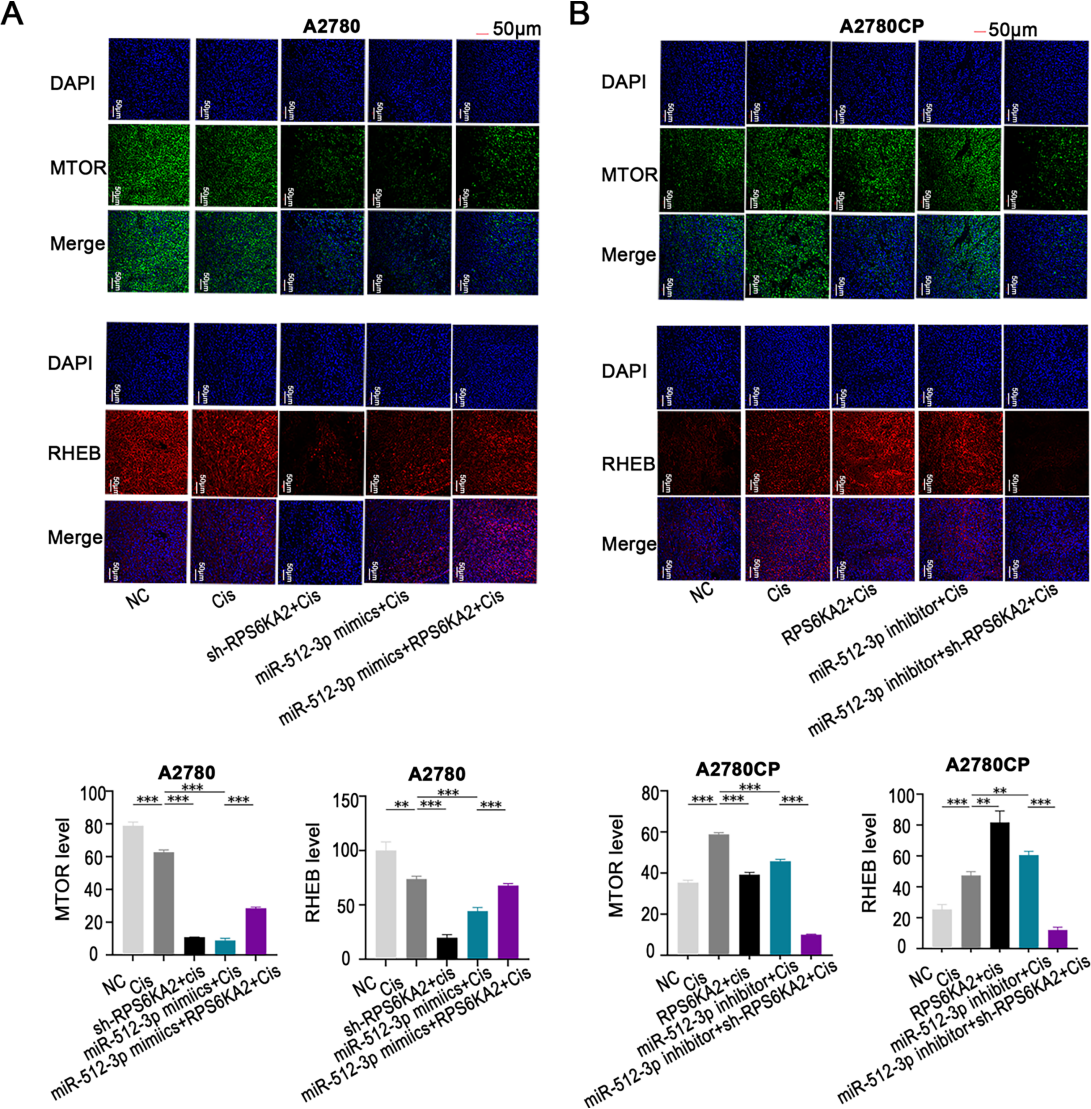
The miR-512-3p/RPS6KA2 axis modulates the autophagy signaling pathway. (**A**) A2780 cells were distributed into five experimental groups: negative control (NC), Cisplatin treatment (Cis), sh-RPS6KA2 + Cis, miR-512-3p mimics + Cis, and miR-512-3p mimics + Cis + RPS6KA2; (**B**) similarly, A2780CP cells were also separated into five groups: NC, cisplatin (Cis), RPS6KA2 + Cis, miR-512-3p inhibitor + Cis, and miR-512-3p inhibitor + Cis + sh-RPS6KA2. Cell immunofluorescence staining was performed to evaluate MTOR and RHEB expression levels. Nuclei were labeled in blue, MTOR signal appears in green, and RHEB is shown in red. **p < 0.01; ***p < 0.001

*In vitro*, treatment with miR-512-3p mimics in combination with cisplatin decreased the expression of MTOR and RHEB. However, when miR-512-3p mimics were co-delivered with an RPS6KA2 overexpression plasmid and cisplatin, the expression of these proteins was restored (Fig. 7A). In contrast, administration of a miR-512-3p inhibitor along with cisplatin increased MTOR and RHEB expression, but this effect was reversed upon addition of sh-RPS6KA2 (Fig. 7B). These findings suggest that miR-512-3p activates the autophagic pathway by directly targeting and downregulating RPS6KA2.

**Figure 8:**
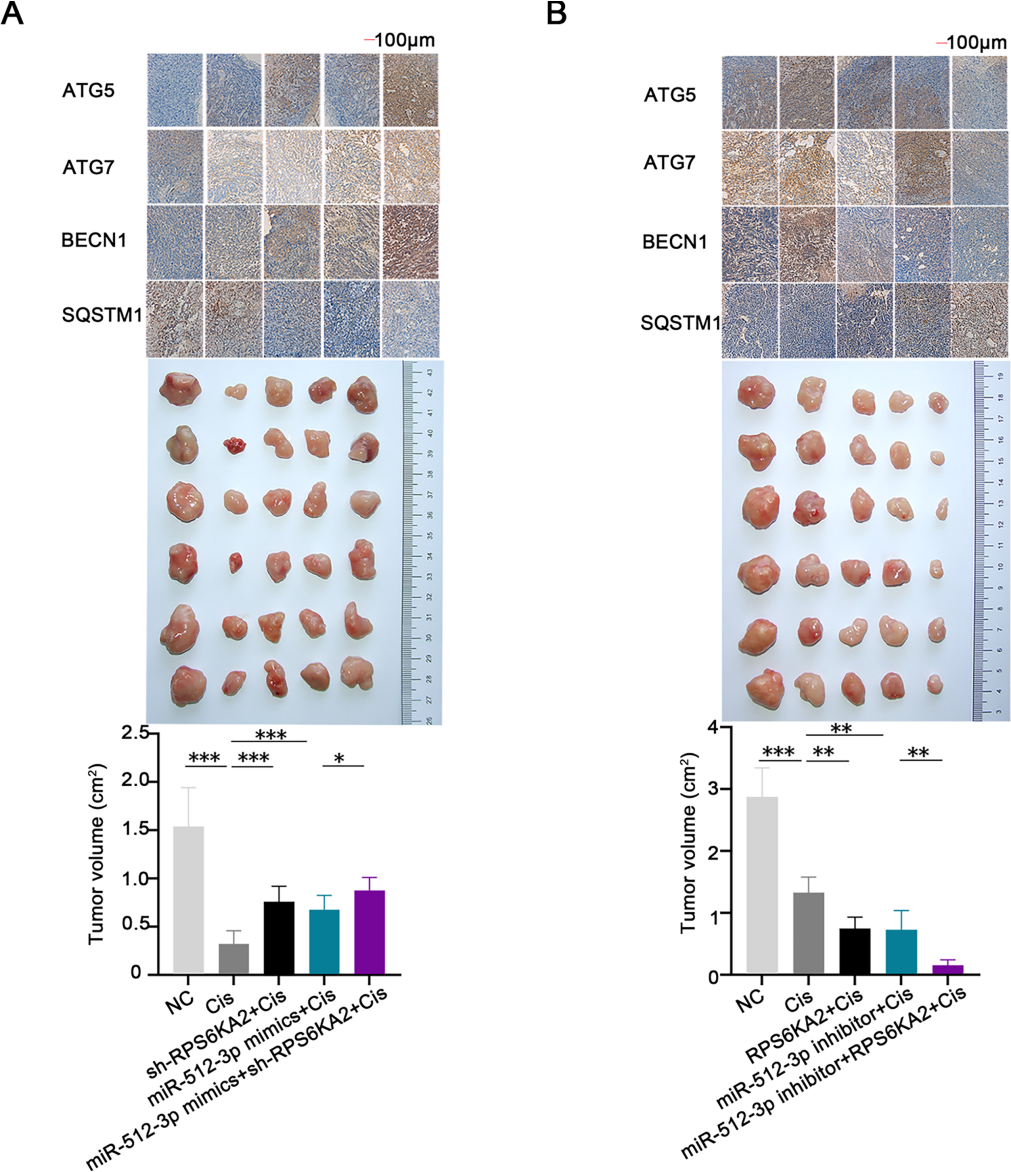
The miR-512-3p/RPS6KA2 axis modulates the autophagy signaling pathway and influences cisplatin resistance in ovarian cancer. (**A**) Subcutaneous xenograft mouse models were allocated into five groups: normal control (NC), Cisplatin treatment (Cis), sh-RPS6KA2 + Cis, miR-512-3p mimics + Cis, and miR-512-3p mimics + Cis + sh-RPS6KA2. Tumor volumes were monitored and compared among groups. Immunohistochemistry analysis was performed to detect the expression levels of ATG5, ATG7, BECN1, and SQSTM1; (**B**) In a parallel experiment, subcutaneous tumor-bearing mice were divided into five groups: NC, Cis, RPS6KA2 + Cis, miR-512-3p inhibitor + Cis, and miR-512-3p inhibitor + Cis + RPS6KA2. Tumor sizes were recorded and compared. Immunohistochemistry was conducted to evaluate the expression of ATG5, ATG7, BECN1, and SQSTM1. *p < 0.05; **p < 0.01; ***p < 0.001

*In vivo*, co-administration of the sh-RPS6KA2 plasmid and cisplatin significantly increased the expression of ATG5, ATG7, and BECN1, as well as promoted subcutaneous tumor growth compared to cisplatin monotherapy (Fig. 8A). The addition of miR-512-3p mimics further amplified these effects (Fig. 8A). In contrast, administration of the RPS6KA2 overexpression plasmid together with cisplatin reduced the expression of ATG5, ATG7, and BECN1 and inhibited tumor expansion (Fig. 8B). Notably, co-treatment with the miR-512-3p inhibitor, RPS6KA2 plasmid, and cisplatin led to even greater reductions in both gene expression and tumor volume (Fig. 8B). Collectively, these results confirm that miR-512-3p enhances autophagic signaling in ovarian cancer by suppressing RPS6KA2 expression.

### 3.7 Relationship between Autophagy and Cisplatin Resistance in Ovarian Cancer

**Figure 9:**
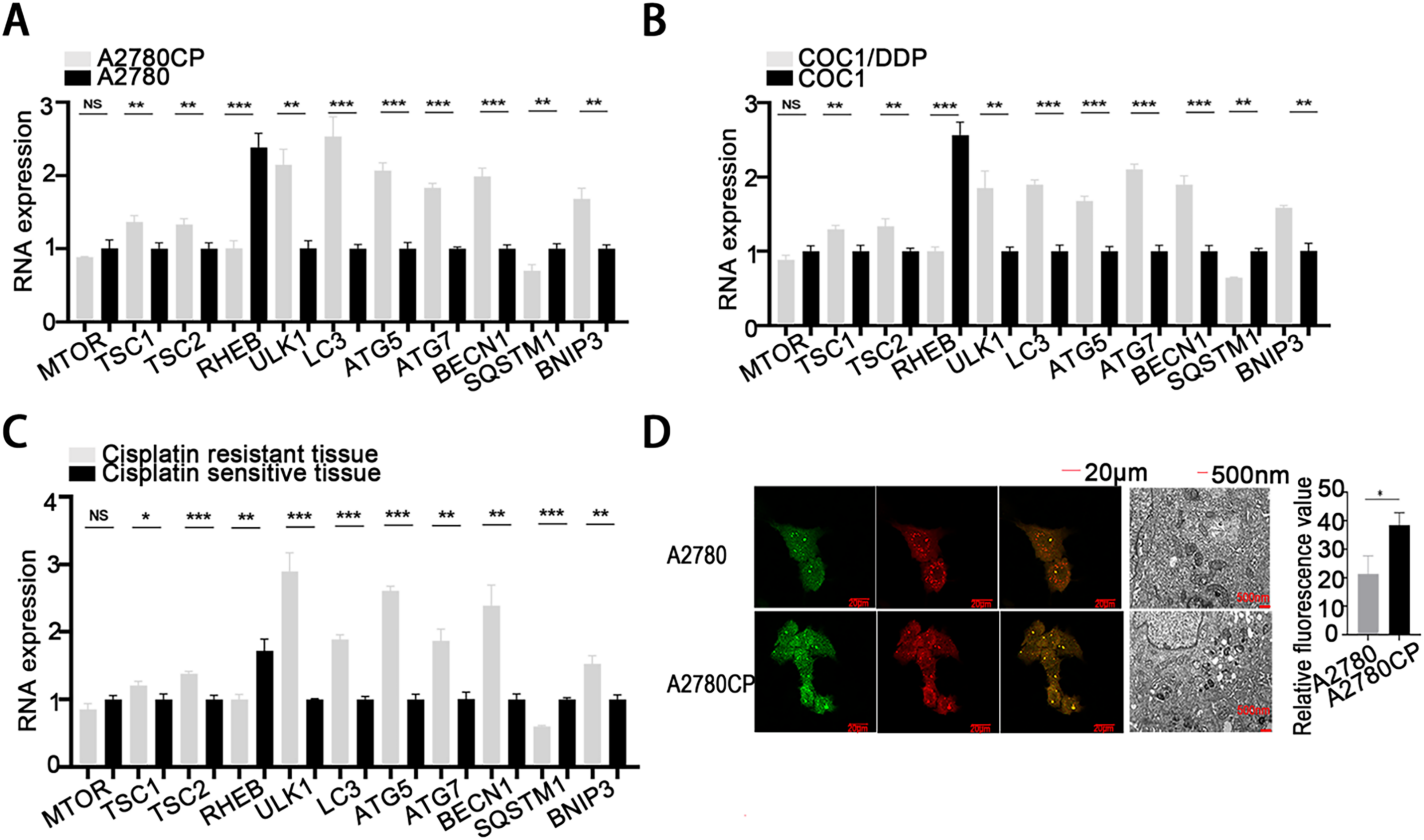

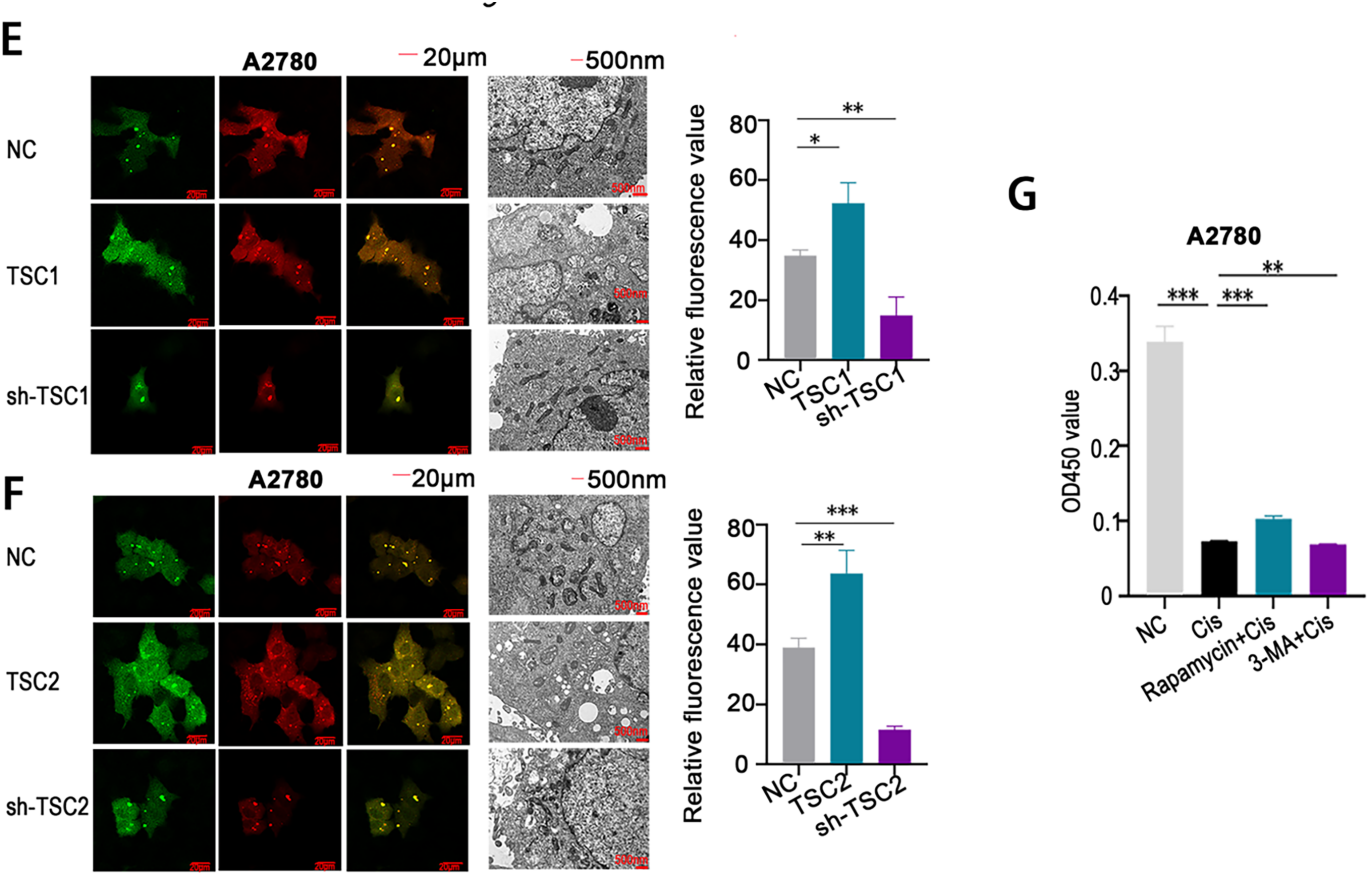
Involvement of autophagy in mediating cisplatin resistance in ovarian cancer. (**A**–**C**) qRT-PCR analysis was conducted to compare the expression levels of autophagy-related proteins in A2780 and A2780CP (**A**), COC1 and COC1/DDP (**B**), as well as in clinical samples from cisplatin-sensitive (n = 3) and cisplatin-resistant (n = 3) ovarian tumors (**C**); (**D**) Autophagic flux and autophagosome formation were assessed using mRFP-GFP-LC3 assays and high-resolution transmission electron microscopy (×10,000 magnification). Green fluorescence indicates autophagosomes, red fluorescence indicates autolysosomes, and yellow fluorescence reflects the early formation stage of autophagosomes; (**E**,**F**) A2780 cells transfected with either TSC1 overexpression plasmid or sh-TSC1 (**E**), and TSC2 overexpression plasmid or sh-TSC2 (**F**), were analyzed through mRFP-GFP-LC3 assays and high-resolution transmission electron microscopy to assess alterations in autophagic flux and autophagosome formation (×10,000 magnification); (**G**) A2780 cells were assigned to four groups: negative control (NC), cisplatin treatment, rapamycin+cisplatin treatment, and 3-MA+cisplatin treatment. Cell viability was determined by CCK-8 assay based on OD_450_ readings. *p < 0.05; **p < 0.01; ***p < 0.001; NS, not significant (*p* > 0.05)

This study conducted a systematic comparison of autophagy-related gene expression profiles between cisplatin-sensitive and cisplatin-resistant ovarian cancer cells. The results showed that genes promoting autophagy, such as TSC1, TSC2, ULK1, LC3, ATG5, ATG7, BECN1, and BNIP3, were markedly upregulated in cisplatin-resistant cells and tissues relative to sensitive ones (Fig. 9A–C). In contrast, RHEB and SQSTM1, known negative regulators of autophagy, showed decreased expression in resistant samples (Fig. 9A–C), indicating a strong correlation between autophagy activation and the development of cisplatin resistance in ovarian cancer.

Further analysis using mRFP-GFP-LC3 fluorescence analysis and high-resolution transmission electron microscopy demonstrated increased autophagic flux and a higher number of autophagosomes in cisplatin-resistant cells compared to sensitive cells (Fig. 9D). Overexpression of TSC1 and TSC2 was shown to induce autophagy, as confirmed by both mRFP-GFP-LC3 analysis and transmission electron microscopy (Fig. 9E,F). In contrast, silencing TSC1 and TSC2 with shRNA vectors significantly reduced autophagic activity (Fig. 9E,F).

Functional experiments demonstrated that co-treatment with an autophagy inducer and cisplatin led to increased cell viability (OD_450_ values) in CCK-8 assays, whereas combining an autophagy inhibitor with cisplatin resulted in reduced viability (Fig. 9G). These results collectively demonstrate that suppression of autophagy enhances cisplatin sensitivity in ovarian cancer cells, highlighting autophagy modulation as a promising therapeutic approach for overcoming cisplatin resistance in ovarian cancer.

### 3.8 Targeting RPS6KA2 Therapy Enhances the Sensitivity of Ovarian Cancer Cells to Cisplatin

**Figure 10:**
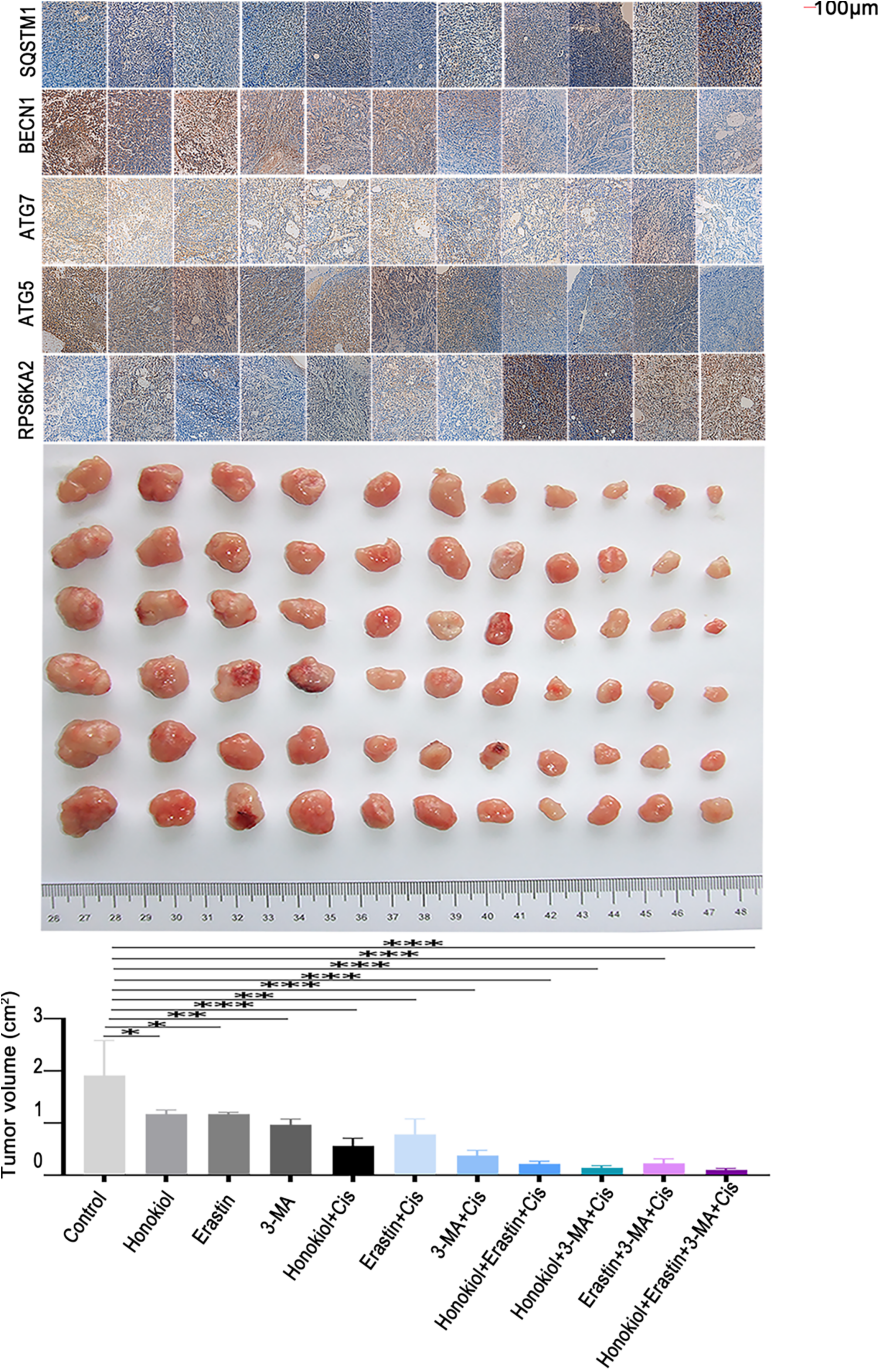
Targeting RPS6KA2 improves cisplatin sensitivity in ovarian cancer. A subcutaneous xenograft mouse model was established and animals were randomly assigned to 11 treatment groups receiving various interventions, including an RPS6KA2-targeted drug (Honokiol), a ferroptosis inducer (Erastin), and an autophagy inhibitor (3-MA). Tumor volumes were monitored over time and compared among the groups. Immunohistochemical analysis was also performed to evaluate alterations in the expression of RPS6KA2 and autophagy-related proteins. *p < 0.05; **p < 0.01; ***p < 0.001

**Figure 11:**
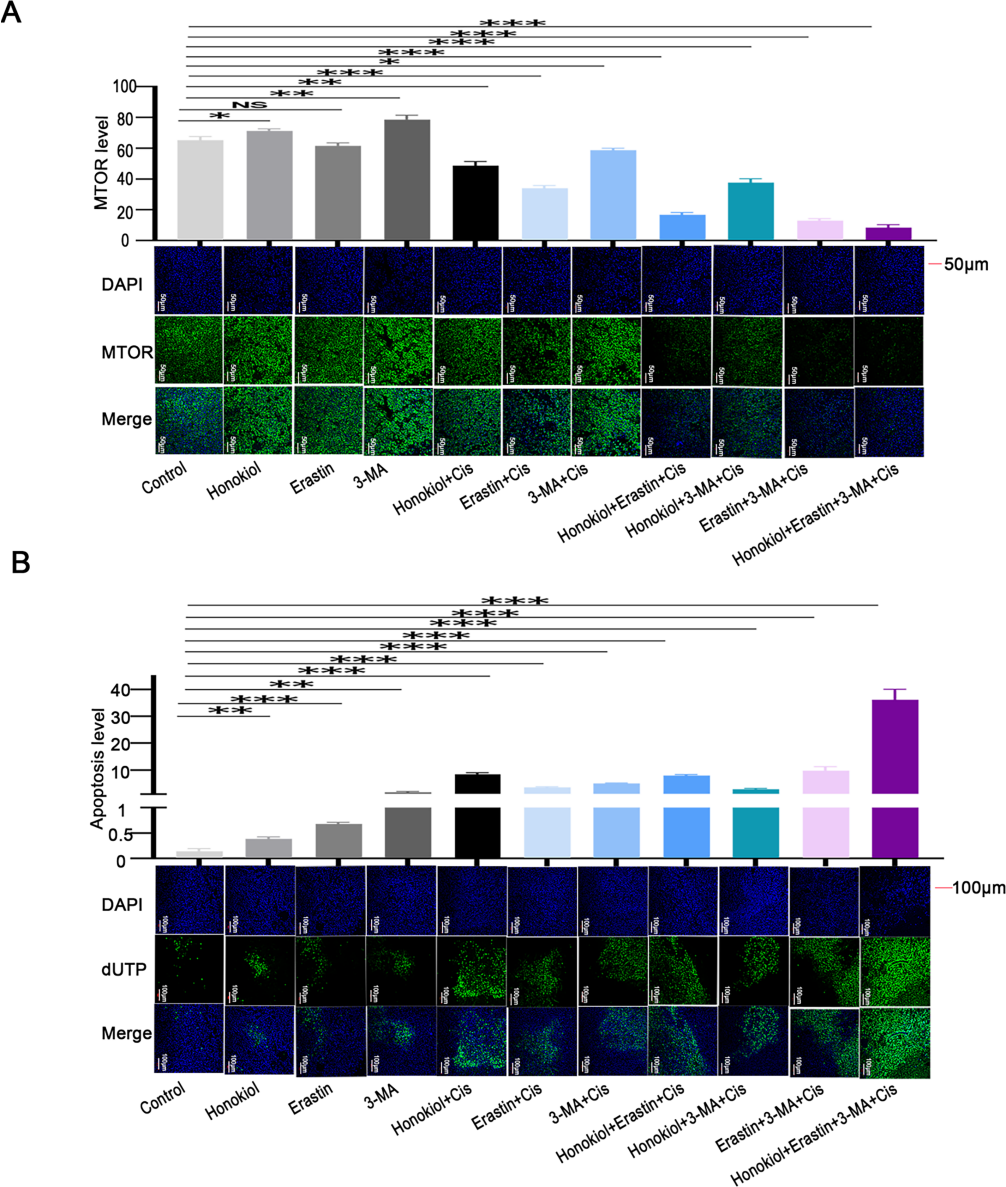
Impact of RPS6KA2 targeting on MTOR expression and apoptosis in ovarian cancer cells. Tumor-bearing mice were randomly distributed into 11 groups and administered various agents, including an RPS6KA2-targeted drug (Honokiol), a ferroptosis inducer (Erastin), and an autophagy inhibitor (3-MA). Immunofluorescence staining was performed to examine changes in MTOR expression across groups; (**A**) nuclei are labeled in blue, and MTOR signal is shown in green. (**B**) TUNEL analysis was conducted to assess apoptotic levels among groups; blue fluorescence represents the nucleus, and green fluorescence marks apoptotic cells. *p < 0.05; **p < 0.01; ***p < 0.001; NS, not significant (*p* > 0.05)

Honokiol has been previously shown to enhance ERK1/2 phosphorylation [34]. Since RPS6KA2 acts as a downstream substrate of the ERK1/2 signaling cascade and is positively regulated by this pathway [35], Honokiol was employed in this study as a potential activator of RPS6KA2. *In vivo* results revealed that, compared to control group, Honokiol treatment significantly upregulated the expression of RPS6KA2, SQSTM1 and mTOR, while simultaneously reducing levels of key autophagy-related proteins (ATG5, ATG7, BECN1). This was accompanied by a marked decrease in tumor volume and an increase in apoptosis in ovarian cancer models (Figs. 10 and 11A,B). Additionally, administration of the autophagy inhibitor 3-methyladenine (3-MA) led to increased MTOR expression and downregulation of ATG5, ATG7, and BECN1, resulting in reduced tumor volume and elevated apoptosis cell death. Similarly, treatment with the ferroptosis activator (Erastin) not only suppressed tumor volume but also decreased the expression of autophagy-related proteins (ATG5, ATG7, and BECN1), along with increased cell apoptosis (Figs. 10 and 11A,B). Notably, the most robust antitumor effect was observed when Honokiol, 3-MA, Erastin, and cisplatin were administered in combination, leading to significantly greater tumor inhibition (Fig. 10). These findings indicate that targeting RPS6KA2, particularly in conjunction with modulation of autophagy and ferroptosis, may represent a promising therapeutic approach for ovarian cancer.

## 4 Discussion

This study reveals that RPS6KA2 enhances the sensitivity of ovarian cancer cells to cisplatin by suppressing the autophagy signaling pathway, representing a novel and significant contribution to the field. As a critical ribosomal protein kinase, RPS6KA2 serves as a key effector in the mitogen-activated protein kinase (MAPK) cascade, where it is activated through phosphorylation of ERK1/2. The MAPK pathway is essential for relaying extracellular signals from the cell membrane to the nucleus, thereby regulating gene expression and cytoplasmic activities. Prior research has indicated that impaired MAPK signaling pathway is closely linked to platinum resistance in ovarian cancer [36,37]. Notably, RPS6KA2 also functions as a central regulator within the mechanistic target of rapamycin (mTOR) signaling pathway, which negatively regulates autophagy by preventing the interaction of TSC1 and TSC2. mTOR is a pivotal eukaryotic kinase that controls fundamental cellular processes such as cell growth, apoptosis, and autophagy by coordinating protein synthesis and transcriptional regulation. Accumulating evidence suggests that activation of the autophagy contributes significantly to cisplatin resistance in ovarian cancer, making this pathway a promising target for therapeutic intervention to counteract chemoresistance [38]. Collectively, these insights underscore the pivotal role of RPS6KA2 in mitigating cisplatin resistance through modulation of key signaling networks in ovarian cancer.

Furthermore, this study reveals that RPS6KA2 enhances ferroptosis, indicating that modulation of ferroptosis represents an additional key mechanism by which RPS6KA2 regulates cisplatin sensitivity in ovarian cancer cells. Accumulating evidence has established that the inhibition of ferroptosis is strongly associated with cisplatin resistance in ovarian cancer [39]. Prior research has demonstrated that mex-3 RNA binding family member A (MEX3A) contributes to tumor progression by regulating p53 degradation, thereby suppressing ferroptosis [40]. Similarly, acyl-CoA synthetase long chain family member 1 (ACSL1) acts as a negative regulator of ferroptosis and increases chemoresistance by up-regulating ferroptosis suppressor 1 (FSP1) in ovarian cancer cells [41]. GPX4, a central inhibitor of ferroptosis, was found to be overexpressed through Frizzled-7 (FZD7)-mediated activation of the oncogene TP63 and the glutathione metabolic pathway, ultimately reducing cisplatin-induced cell death [42]. Conversely, treatment with shikonin was shown to trigger ferroptosis by inducing heme oxygenase 1 (HMOX1) expression, thereby sensitizing ovarian cancer cells to cisplatin [43]. Collectively, these findings provide strong external support for the central hypothesis of this study, reinforcing the role of RPS6KA2 in modulating ferroptotic pathways to influence therapeutic response.

The inhibition of ferroptosis and activation of autophagy are strongly associated with cisplatin resistance in ovarian cancer. Evidence from prior research indicates that can trigger the macroautophagic/autophagic degradation of, thereby significantly promoting ferroptosis [44]. Additionally, studies have demonstrated that genetic ablation of ATG5 or ATG7 diminishes ferroptosis by reducing intracellular iron accumulation and lipid peroxidation. Lipocalin 2, a gene implicated in ferroptosis, has been shown to modulate macroautophagy/autophagy and enhance oxidative stress-induced ferroptosis [45]. Collectively, these observations support the emerging view that ferroptosis may be classified as a type of autophagy-dependent cell death [46,47]. However, the exact nature of the interaction between autophagy and ferroptosis in mediating cisplatin resistance in ovarian cancer is still not completely understood, representing a key limitation of the present study. Future investigations will focus on clarifying the molecular mechanisms underlying this interplay in the context of chemoresistance.

More importantly, this study reveals that targeting RPS6KA2 enhances the responsiveness of ovarian cancer cells to cisplatin. Notably, this effect is further amplified when RPS6KA2 targeting is combined with either autophagy inhibitors or ferroptosis inducers, leading to greater cisplatin sensitivity. Earlier research has indicated that modulating autophagy can impede ovarian cancer cell proliferation, with 3-MA shown to suppress both proliferation and metastasis [48,49]. Additionally, erastin, a known inducer of ferroptosis, has been reported to increase the susceptibility of these cells to cisplatin [50,51]. Despite these advances, the therapeutic potential of combination of 3-MA and Erastin in ovarian cancer remains largely unexplored. Given that cisplatin resistance in ovarian cancer involves multiple interconnected pathways and molecular mechanisms, a multi-targeted therapeutic approach may offer a more effective treatment strategy. The findings presented here strongly support the feasibility and promise of drug sensitization as a novel avenue for improving ovarian cancer therapy.

However, this study is subject to certain limitations. While our findings suggest that RPS6KA2 modulates the cisplatin sensitivity in ovarian cancer cells through the regulation of autophagy and ferroptosis, the exact nature of the crosstalk between these two cellular processes remains unclear. In the preliminary phase of our experiments, luciferase reporter assays were employed as a functional indicator, as they capture the overall impact of miRNA activity—including both mRNA degradation and translational suppression—and align with widely accepted approaches for validating miRNA targets. Nevertheless, this method does not distinguish between these two distinct regulatory mechanisms, either of which may contribute to the function of miR-512-3p. This gap will be systematically investigated in subsequent research.

In conclusion, the miR-512-3p/RPS6KA2 axis plays a critical role in regulating cisplatin sensitivity in ovarian cancer by coordinately influencing both autophagy and ferroptosis signaling pathway at both *in vitro* and *in vivo* levels. Simultaneous targeting of RPS6KA2 and the associated autophagy-ferroptosis signaling networks markedly increases the responsiveness of ovarian cancer cells to cisplatin. These findings not only reveal new mechanistic understanding but also highlight a potential therapeutic approach for enhancing drug sensitivity in the treatment of ovarian cancer.

## Data Availability

All the study data are included in the article.
